# High fidelity sorting of remarkably similar components *via* metal-mediated assembly†
†Electronic supplementary information (ESI) available: See DOI: 10.1039/c5sc01689d



**DOI:** 10.1039/c5sc01689d

**Published:** 2015-06-02

**Authors:** Lauren R. Holloway, Michael C. Young, Gregory J. O. Beran, Richard J. Hooley

**Affiliations:** a University of California – Riverside , Department of Chemistry , Riverside , CA 92521 , USA . Email: richard.hooley@ucr.edu

## Abstract

Subtle differences in ligand coordination angle and rigidity lead to high fidelity sorting between individual components displaying identical coordination motifs upon metal-mediated self-assembly. Narcissistic self-sorting can be achieved between highly similar ligands that vary minimally in rigidity and internal coordination angle upon combination with Fe(ii) ions and 2-formylpyridine. Selective, sequential cage formation can be precisely controlled in a single flask from a mix of three different core ligands (and 33 total components) differing only in the hybridization of one group that is uninvolved in the metal coordination process.

## Abstract 2

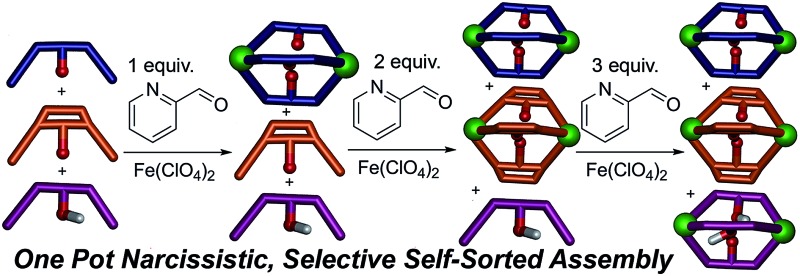
Subtle differences in coordination angle and rigidity lead to narcissistic self-sorting between highly similar individual components upon metal-mediated assembly.

## 

The selective assembly of multiple components into larger structures relies on the sum of a collection of small interactions reaching a stable whole.^1^ A variety of different weak forces can be exploited,^2^ from hydrogen bonds and London dispersion forces,^2^ to more exotic interactions such as halogen bonds^3^ and π–π/CH–π forces, among others.^4^ In addition, solvation effects are a vital component of all assembly and recognition processes, especially in aqueous solution.^5^ All these weak forces are the inspiration for, and have been exploited towards the creation of a variety of synthetic self-assembled complexes.^6–8^ Weak, reversible transition metal–ligand interactions have proved a fruitful tool for the controlled self-assembly of different structures, from simple polygons^9,10^ to polyhedra,^11–13^ intertwined catenanes^14^ and molecular switches.^15^ As the structures become more complex, greater control of the assembly process is necessary, and self-sorting between different ligand types becomes paramount. Obviously, when constructing a complex assembly, the larger the energetic difference between each assembled outcome, the more effective the sorting. Varying the type of metal–ligand contact, ligand size, denticity and coordination angle^16–19^ are all effective strategies to control self-sorting in complex assemblies.^20^ If the components are too similar, though, a complex mixture of species can result, either of stereoisomers^21^ or multiple stoichiometries of assembly.^22,23^ Selective assembly of these components is known as self-sorting, and this process falls into one of two general categories. Narcissistic self-sorting involves selective complex formation with a single ligand type,^24–29^ whereas social self-sorting describes the selective incorporation of different ligand types in a single assembly.^30–37^ Both types of self-sorting are known in metal–ligand and H-bonded self-assemblies,^38^ but the majority of studies focus on maximal discrimination between varied ligand cores.

Subtle differences between otherwise identical ligands can have large effects on an assembly, however. As part of our ongoing studies of the control of self-assembly by the application of functional groups to a central ligand core,^39–42^ we have observed that small differences in functionality can have drastic effects on self-sorting behavior.^42^ Introducing hydrogen bonding groups can bias the outcome of metal–ligand mediated assembly,^39^ and pendant functional groups uninvolved in the coordination process can affect sorting and assembly in multiple ways.^41^ Here, we show that small variations in ligand geometry and rigidity can effect high fidelity narcissistic self-sorting on the assembly of Fe_2_L_3_ mesocate structures.

We targeted a self-assembling system that can be easily varied in small increments *via* similar synthetic routes, allowing control of individual variables. Fe^II^-mediated multicomponent self-assembly of diaminosuberone and xanthone-based ligands satisfies these conditions: they can be easily varied and the assembled complexes are relatively simple to synthesize and amenable to rapid characterization by NMR methods. Small variations are possible without large changes to the overall structure, and flexibility is minimized, ensuring that the assemblies form with identical stoichiometries. The four main ligand cores used for this study are shown in Fig. 1. Cores **A–C** are all easily accessed from dibenzosuberone *via* selective dinitration followed by partial (**A**) or exhaustive reduction (**B**). The suberenone core **C** is accessible from 3,7-dinitrosuberone *via* benzylic radical bromination, dehydrohalogenation and reduction to the diamine. Xanthone core **D** was synthesized from xanthone using the same route as for **A**.

**Fig. 1:**
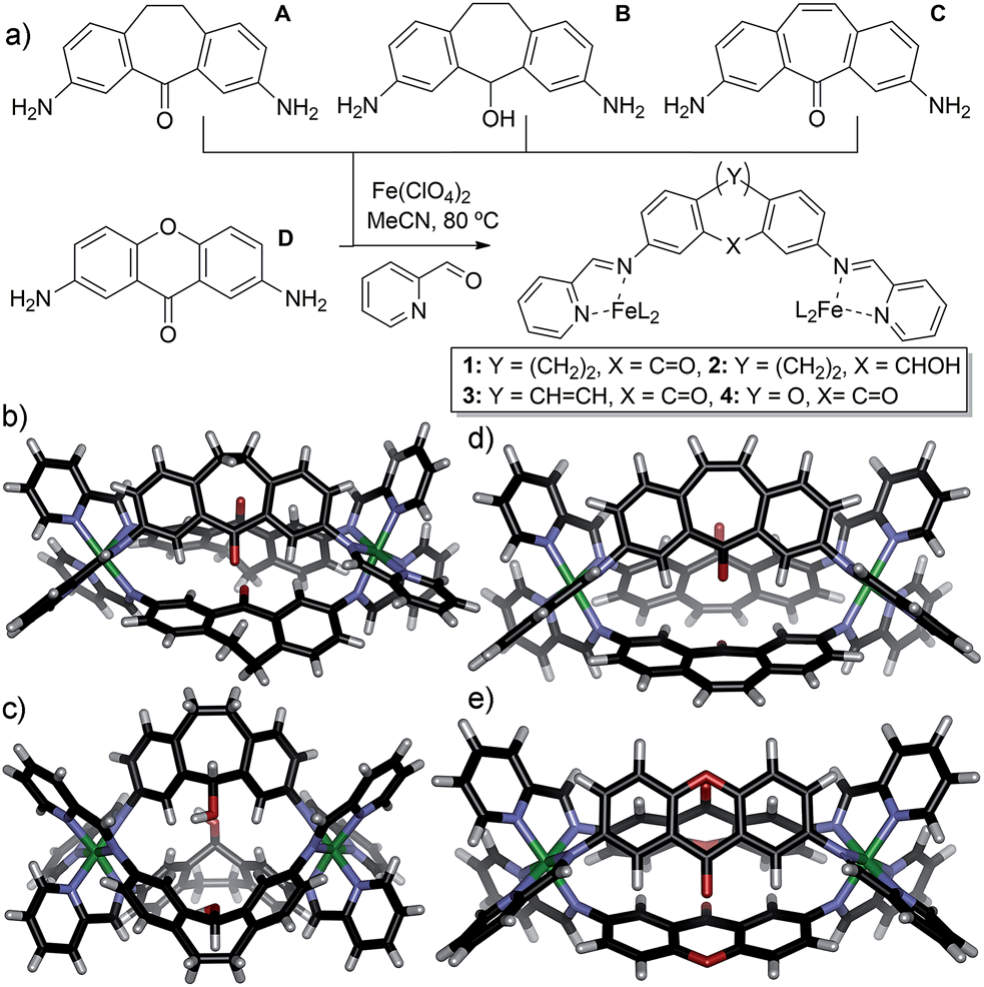
(a) Ligands used and self-assembly process; (b) crystal structure of cage **1**; (c) crystal structure of cage **2**; (d) DFT minimized structure of cage **3**; (e) DFT minimized structure of cage **4**.

The four cores are geometrically quite similar, and all four are capable of forming M_2_L_3_ mesocate cage complexes **1–4** (Fig. 1) upon treatment with 2-formylpyridine (PyCHO) and Fe(ClO_4_)_2_ in acetonitrile solvent. Each ligand core has a slightly V-shaped structure and leads to the entropically favored M_2_L_3_ stoichiometry upon assembly, as opposed to more linear ligands^13,43^ that form higher stoichiometry aggregates. These complexes have many similarities: there are no large differences such as overall charge, stoichiometry or variable metal coordinating groups. In addition, there are no large steric differences between the internal groups: **A**, **C** and **D** all contain carbonyl functions, and the alcohol in **B** is of the same size (although provides different hydrogen bonding characteristics). The variability between these core ligands comes only from the rigidity of the backbone: suberenone **C** is fully conjugated and nearly planar, as is xanthone ligand **D**. Suberone **A** has mildly flexible CH_2_ groups at the back of the ligand, and suberol **B** has a 109° out of plane bend due to the sp^3^ centers.

To test the selectivity behavior of the assembly process, pairs of ligands (1 equivalent each) were combined with two equivalents PyCHO and 0.67 equivalents Fe(ClO_4_)_2_ in CD_3_CN, amounts sufficient to form one favored cage complex only. The ^1^H NMR spectra for the combination of suberone **A** and suberenone **C** are shown in Fig. 2. After initial mixing and a short stir, the characteristic purple color of an Fe^II^-iminopyridine complex was observed, but only non-discrete aggregates were present in the ^1^H NMR spectrum (Fig. 2a). After heating at 80 °C for 8 h, however, the spectrum sharpened and discrete species could be observed (Fig. 2b). Encouragingly, only two sets of peaks were visible, those of suberone cage **1** and suberenone ligand **C**. The assembly process showed complete selectivity for suberone self-assembly: not only was the suberone cage **1** formed exclusively over suberenone cage **3**, no heterocomplexes were observed. No peaks for PyCHO were observed either, indicating that all aldehyde has been consumed and the only observable species is the cage complex. When an excess of PyCHO and Fe(ClO_4_)_2_ was added to this mixture, a complex NMR spectrum including cage **1** and broad peaks formed: after another 8 h heat at 80 °C, the spectrum sharpened again to give peaks for the two cages alone. The sorting experiment was repeated for the other combinations of ligands (see ESI† for full spectral data). In the case of ligands **A–C**, all derived from the suberone scaffold, the same combination of both ligand-selective and narcissistic self sorting was observed. In the presence of sufficient PyCHO and Fe(ClO_4_)_2_ to form one complex, the only peaks observable by ^1^H NMR analysis belonged to a single cage complex and free ligand. These endpoints indicate that the order of selectivity for cage assembly is suberone **A** > suberenone **C** > suberol **B**. Even though the coordinating iminopyridine motifs are the same and the coordination geometries similar, clear selectivity was observed.

**Fig. 2:**
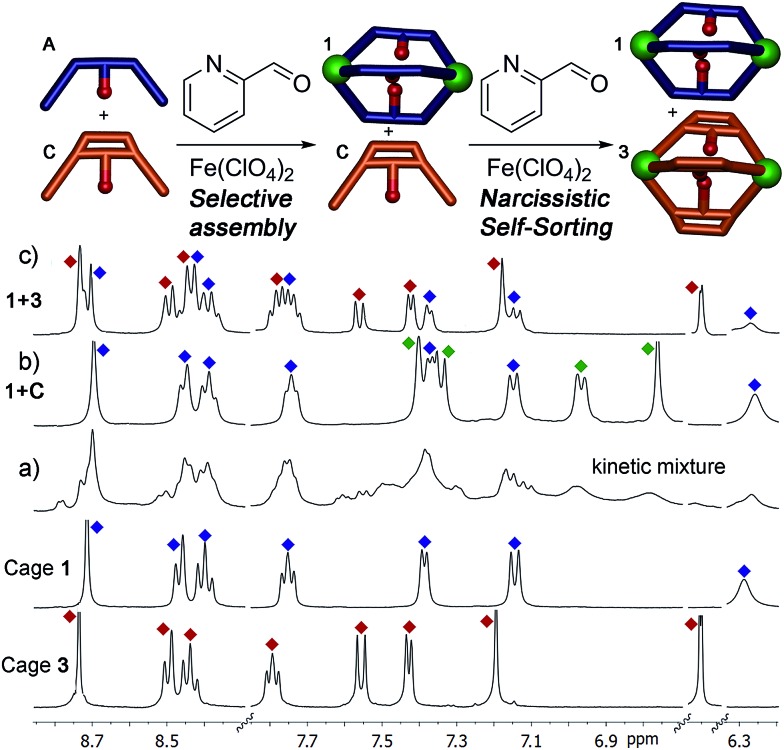
Self-assembly of core ligands **A** and **C**. Downfield regions of the ^1^H NMR spectra obtained (a) immediately after first addition of 0.67 eq. Fe(ClO_4_)_2_ + 2 eq. PyCHO at 298 K; (b) after heating (8 h@353 K); (c) after second addition 0.67 eq. Fe(ClO_4_)_2_ + 2 eq. PyCHO and heating (8 h, 353 K); spectra of pure cages **1** and **3** (400 MHz, CD_3_CN, 298 K, [**A**] = [**C**] = 36 mM). Blue = cage **1**; red = cage **3**; green = ligand **C**.

The differences between ligand and cage peaks were easily distinguishable by diffusion analysis. 2D DOSY spectra were taken of the cage/ligand endpoint samples (*e.g.* that shown in Fig. 2b between cage **1** and ligand **C**), and the cages and ligands showed large differences in diffusivity as shown in Fig. 3. For example, cage **1** showed a diffusion constant of 8.39 × 10^–10^ m^2^ s^–1^, whereas ligand **C** diffused at 2.15 × 10^–9^ m^2^ s^–1^. There was little difference between the diffusivity of individual cages **1–3** or ligands **A–C**, as might be expected given their similar structures. ESI-MS analysis also corroborated the sorting. The ESI samples were formed by combining equimolar amounts of ligands **A** and **C** in CD_3_CN, followed by addition of 2 equivalents PyCHO and 0.67 equivalents Fe(ClO_4_)_2_ and heating for 8 h at 80 °C. ESI-MS analysis showed only M^+^ peaks for the cage **1** ions ([**1**·(ClO_4_)_2_]^2+^ and [**1**·(ClO_4_)]^3+^), with no ions for heterocomplexes or cage **3** present. Other combinations of **A–C** were tested, and the results were the same, showing no ions corresponding to heterocomplexes.

**Fig. 3:**
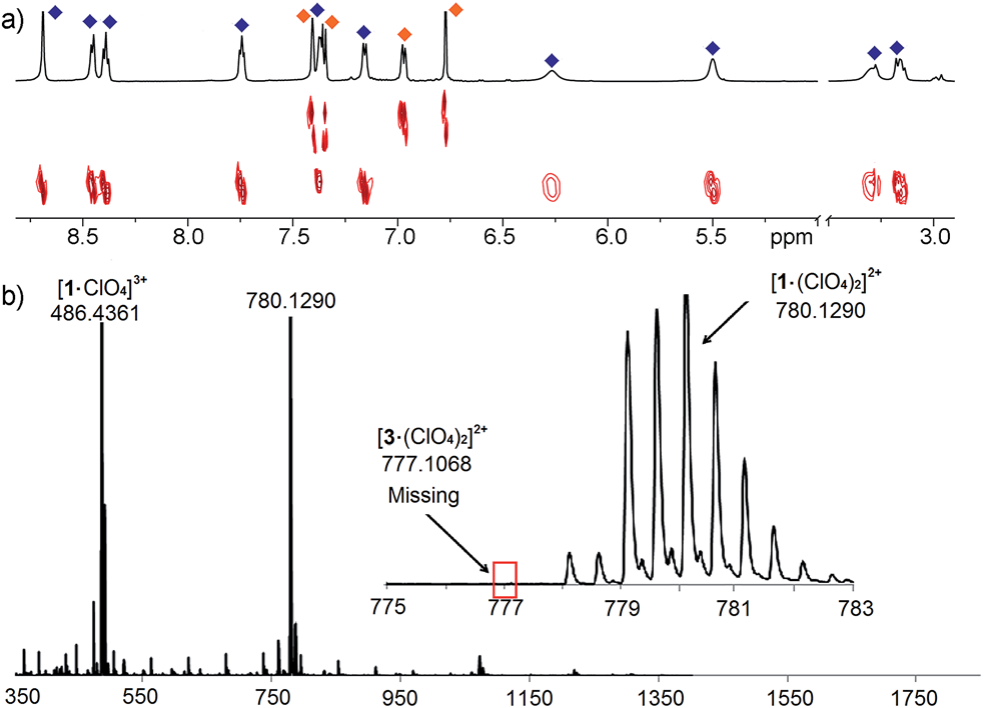
(a) 2D DOSY spectrum of cage **1** and ligand **C** observed after addition of 0.67 eq. Fe(ClO_4_)_2_ + 2 eq. PyCHO to an **A**/**C** mix and 8 h heating@353 K (600 MHz, CD_3_CN, 298 K, blue = cage **1**; orange = ligand **C**); (b) ESI-MS spectrum, indicating only the presence of cage **1**.

Most excitingly, the self-sorting fidelity was strong enough to allow discrimination of all three components in the same system, as shown in Fig. 4. The three suberone-derived ligands **A**, **B** and **C** were combined in equimolar amounts (1 equivalent, 14 mM) in CD_3_CN, followed by addition of 2 equivalents PyCHO and 0.67 equivalents Fe(ClO_4_)_2_ in CD_3_CN. As can be seen in Fig. 3c, only suberone cage **1** is formed, and the suberol and suberenone ligands **B** and **C** remain in solution. If another aliquot of PyCHO/Fe(ClO_4_)_2_ is added (Fig. 3b), the suberone cage **1** remains intact and the suberenone ligand **C** is converted to cage **3**, leaving the suberol dianiline behind. A final equivalent of PyCHO/Fe(ClO_4_)_2_ confers complete assembly on the system, and again *no heterocomplexes are formed*. Each of these ligands has an identical coordinator, similar coordination geometries and the possibility of forming aggregates of variable stoichiometry (*e.g.* M_4_L_6_), not to mention the possibility of metal-based isomers.^13,21,39,41^ Subtle modification of the rigidity or out of plane bending angle of the ligand backbone is sufficient to confer selective sorting. Each assembly is formed from 11 components in one process, and yet complete fidelity is possible: no mixed complexes are formed, and the assembly can be precisely controlled in a single flask.

**Fig. 4:**
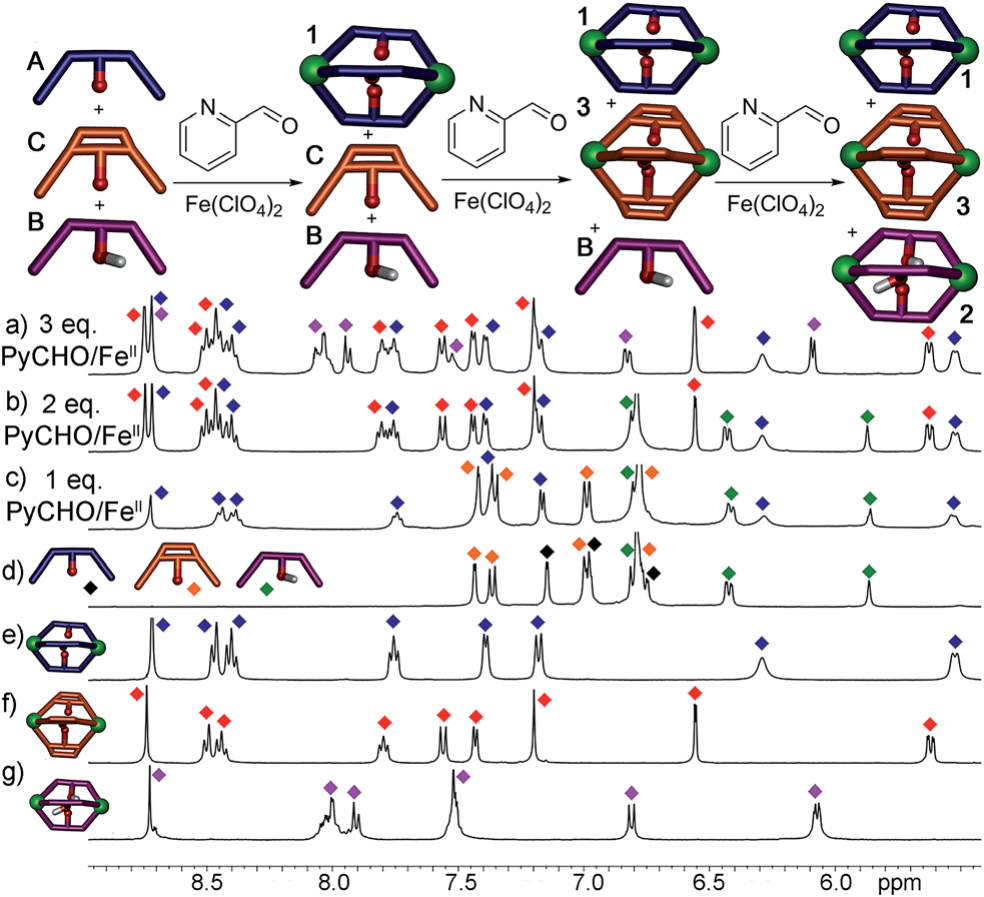
Downfield regions of the ^1^H NMR spectra obtained after addition of 0.67 eq. Fe(ClO_4_)_2_ + 2 eq. PyCHO to a mixture of ligands **A**, **B** and **C** and 8 h heating@353 K; (a) **1** + **2** + **3**; (b) **1** + **B** + **3**; (c) **1** + **B** + **C**; (d) **A** + **B** + **C**. ^1^H NMR spectra of pure cages: (e) **1**; (f) **3**; (g) **2** (400 MHz, CD_3_CN, 298 K). Red = cage **3**; blue = cage **1**; purple = cage **2**; green = ligand **B**; orange = ligand **C**; black = ligand **A**.

While the assembly of the suberone-derived scaffolds was well controlled, selective sorting was not observed when the same experiments were performed with xanthone ligand **D**. Even though the coordination angle difference between **D** and **A** is greater than that between **A** and **C**, far less discrimination between the ligands occurs upon self-assembly. In this case, complex (yet sharp) NMR spectra were observed after the addition of either one equivalent or excess PyCHO and Fe(ClO_4_)_2_ to mixtures of **D** and either **A**, **B** or **C**. Peaks for heterocomplexes were clearly observable in all cases. Notably, peaks corresponding to cage **4** homocomplex were present in very small amounts for each of the tests: evidently xanthone self-assembly is far less favorable than that of the suberone-derived scaffolds. It is not immediately obvious why assembly of a xanthone scaffold should be less favorable, but cage **4** displays unusual magnetic characteristics that may be related. Most self-assembled Fe-iminopyridine complexes (including **1–3**)^40^ are fully diamagnetic^8,12,27,39,41^ and show little variation in their ^1^H NMR spectrum upon heating. In contrast, xanthone cage **4** is weakly paramagnetic and shows changes in chemical shift of up to 5 ppm upon heating to 338 K, suggesting that the assembly leads to a less strongly coordinating environment around each Fe center.

This system is thermodynamically driven: the sorting does not depend on kinetic trapping. As can be seen in Fig. 2a, the system must be heated until the requisite assembled species is formed, and incomplete reaction leads not to a discrete, alternate assembly product, but to an undefined mixture of aggregates. Further heating of the mixtures above led to no change in their ^1^H NMR spectra, even after 24 h at 80 °C. To provide more quantitative data on the process, ligand displacement experiments were performed on the premade cages. One molar equivalent of isolated cage (*e.g.* **1**) was added to 3 equivalents of a different dianiline ligand (*e.g.* **B**) in CD_3_CN and the samples were heated at 50 °C for 8 h. Under anhydrous conditions, the ligand displacement reaction is quite slow: when suberone ligand **A** was added to suberenone cage **3**, only slight changes to the ^1^H NMR spectrum were observed, even after extensive heating (Fig. 5c). This, at first glance, contradicts the results shown above. However, the ligand:PyCHO:Fe^II^ assembly process is not anhydrous, as six equivalents of water are generated during the reaction. When the displacement experiments were repeated in CD_3_CN with the addition of 6 equivalents H_2_O, the isolated cages were completely converted to the more stable homocomplex assemblies after just 1 hour of heating at 50 °C, in an order completely consistent with the stability order shown above. No heterocomplexes were seen in any case.

**Fig. 5:**
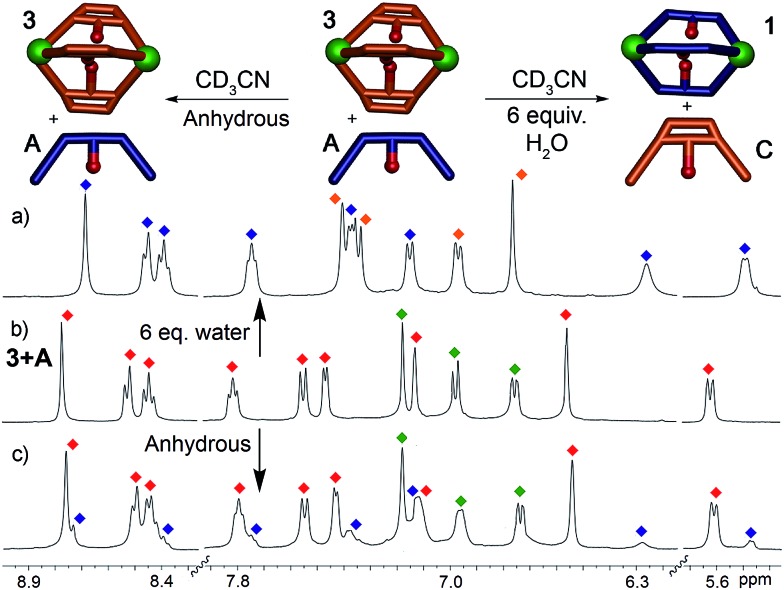
Effect of H_2_O on displacement. ^1^H NMR spectra of the cage **3**: ligand **A** mixture after heating in CD_3_CN in the (a) presence (50 °C, 1 h) and (c) absence (50 °C, 8 h) of 6 molar equivalents of H_2_O (400 MHz, CD_3_CN, 298 K). Red = cage **3**; blue = cage **1**; green = ligand **A**; orange = ligand **C**.

When suberone cage **1** was mixed with suberol dianiline **B** and suberenone dianiline **C**, no change in the spectrum of the cage was observed as expected. When suberol cage **2** was mixed with ligand **A**, peaks for cage **1** and displaced ligand **B** were observed after heating, and addition of suberenone **C** led to the formation of cage **3** and displaced ligand **B**. When suberenone cage **3** was mixed with suberone dianiline **A**, the spectrum changed to that of cage **1** and free suberenone dianiline **C**, but when cage **3** was mixed with dianiline **B**, the spectrum remained unchanged, as expected. The water-accelerated ligand exchange process was amenable to elevated temperature NMR analysis: displacement of the suberenone diamines from cage **3** by ligand **A** is shown in Fig. 6.

**Fig. 6:**
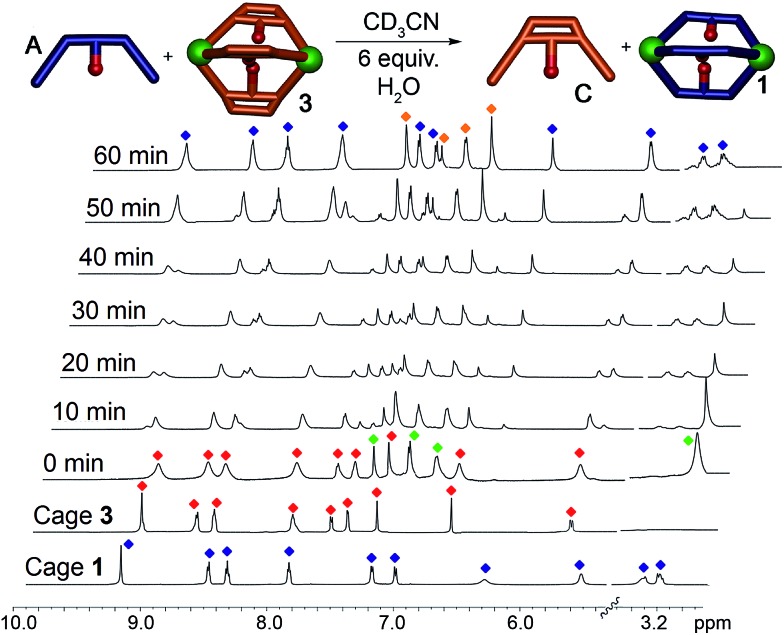
Displacement Experiments. ^1^H NMR spectra of the displacement reaction between cage **3** and dianiline **A** in the presence of 6 equivalents H_2_O (CD_3_CN, 600 MHz, 343 K). Blue = cage **1**; red = cage **3**; green = ligand **A**; orange = ligand **C**.

The spectra show gradual loss of cage **3** peaks and their replacement by freshly formed cage **1**, and no peaks for heterocomplexes are observed during the experiment. No peaks for PyCHO are observed either: the displacement mechanism must involve transimination (as has been observed for other amine additions to Fe-iminopyridine cages^43^) rather than hydrolysis/cage reformation. While heterocomplexes must be present as transient intermediates, they are evidently far less stable than the homocomplexes, exist only fleetingly and cannot be observed at NMR concentrations. The rate increase in the presence of water is interesting, especially in light of the fact that no free aldehyde is observed at any appreciable concentration. Under the concentrations used, water is unlikely to participate in displacement of the imine, and the acceleration is likely due to the formation of small concentrations of Brønsted acid that aids the decomplexation of the iminopyridine ligand from the Fe center and subsequent transimination.

The order of selectivity is quite clear: suberone cage **1** is the most favorable, followed by suberenone **3** and suberol **2**. Xanthone cage **4** can be formed, but is substantially less accessible than the others. In addition, heterocomplexes from ligands **A–C** were not observed, although some mixing was observed with **D**. The question is: why? The major variation between ligands is their rigidity and deformation upon complex formation: there are two relevant angles that can be considered to illustrate these changes. The coordination angle (2*Θ*_c_, Fig. 7) of the four ligands **A–D** and the corresponding cages **1–4** is the major variable here. 2*Θ*_c_ for ligands **A–D** was measured as 93.22°, 87.87°, 86.75° and 125.18° respectively from their minimized structures. The corresponding angles in the self-assembled complexes were also determined (from the crystal (**1**, **2**) or minimized (**3**, **4**) structures). Cage **1** has a coordination angle of 99.89° while that of cage **2** was measured at 69.96°. Cages **3–4** had angles of 95.44° and 115.08° respectively. Changes in coordination angle of between 7 and 17 degrees occur upon assembly, with cage **2** undergoing the greatest angle change. The out-of-plane distortion angle (2*Θ*_b_) also varies, and was defined as the angle between the two terminal anilines and the internal carbon of the central ring. 2*Θ*_b_ for ligands **A–D** were 142.81°, 130.58°, 133.20°, and 174.75°, respectively. Upon cage formation, the ligands become distorted in order to accommodate the geometry of the complex. The ligand cores of cages **1–4** bend at angles of 160.51°, 106.03°, 147.02° and 156.66°, respectively. The degree of out-of-plane distortion caused by cage formation is 17.7°, 24.55°, 13.82°, and 18.09° for cages **1–4**. The metal–metal distances also vary between the cages: the Fe–Fe distances measured from the crystal structures of **1** and **2** are 11.65 Å and 9.70 Å, respectively. The DFT minimized Fe–Fe distances in **3** and **4** are 11.59 Å and 11.69 Å, respectively. The variance in these distances agrees with the observed angle changes: suberol cage **2** is quite distinct from the others, whereas there is little difference between the suberone/suberenone and xanthone cages **1**, **3** and **4**. These structural changes upon assembly provide some explanation for why narcissistic self-sorting occurs and heterocomplexes are not observed: the different ligands are variably deformed upon coordination, and mismatches are not tolerated. Even though the changes are small, the assembly process is able to discriminate between them effectively.

**Fig. 7:**
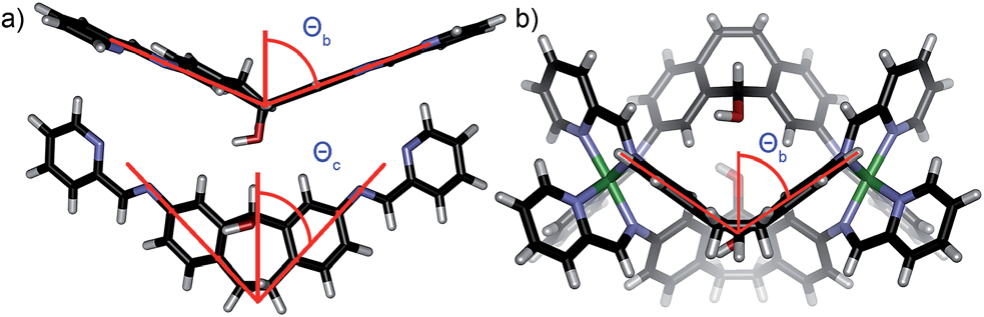
(a) Coordination angle *Θ*_c_ and out of plane bending angle *Θ*_b_ for ligand **B** (2*Θ*_b_ (**B**) = 130.58°); (b) out of plane bending angle *Θ*_b_ for cage **2** (2*Θ*_b_ (**2**) = 106.03°).

In conclusion, we have shown that self-assembly processes can be selectively controlled by small variances in ligand structure. Narcissistic self-sorting between highly similar ligands is observed upon combination with Fe(ii) ions and 2-formylpyridine, and this sorting is correlated to the amount of ligand deformation upon complex formation. Small changes in ligand rigidity between species as similar as suberone, suberenone and suberol scaffolds can be detected by the assembly process, and no heterocomplexes are seen, despite the fact that the coordinating iminopyridine motifs are identical. The discrimination is sufficiently strong to allow sequential formation of the individual cage complexes from a single pot reaction. Further studies on the application of the phenomena to the creation of functional cage complexes are underway in our laboratory.
