# Supplementary material for: High fidelity sorting of remarkably similar components via metal-mediated assembly
Source: Chem Sci. 2015 Jun 2;6(8):4801–6. doi: 10.1039/c5sc01689d (PMC5502402; doi:10.1039/c5sc01689d)
Supplement: Supplementary file 1 [file SC-006-C5SC01689D-s001.pdf]

Electronic Supplementary Material (ESI) for Chemical Science.
This journal is © The Royal Society of Chemistry 2015

High Fidelity Sorting of Remarkably Similar Components via Metal-Mediated Assembly

Lauren R. Holloway, Michael C. Young, Gregory J. O. Beran and Richard J. Hooley*

Department of Chemistry, University of California, Riverside, CA 92521.

richard.hooley@ucr.edu

Electronic Supplementary Information

Table of Contents

General Information 2
Synthesis of Compounds 2
NMR Spectral Data 5
Mass Spectral Data 11
Assembly Mixing Experiments 12
Ligand Displacement Experiments 22
References 24

General Information:

^{1}H and ^{13}C NMR spectra were recorded on a Varian Inova 400 MHz or Varian Inova 500 MHz NMR spectrometer. DOSY spectra were recorded on a Bruker Avance 600 MHz spectrometer. Proton (^{1}H) chemical shifts are reported in parts per million (δ) with respect to tetramethylsilane (TMS, δ=0), and referenced internally with respect to the protio solvent impurity. Deuterated NMR solvents were obtained from Cambridge Isotope Laboratories, Inc., Andover, MA, and used without further purification. Mass spectra were recorded on an Agilent 6210 LC TOF mass spectrometer using electrospray ionization with fragmentation voltage set at 115v and processed with an Agilent MassHunter Operating System. All other materials were obtained from Aldrich Chemical Company, St. Louis, MO, or TCI, Tokyo, Japan and were used as received. Solvents were dried through a commercial solvent purification system (Pure Process Technologies, Inc.). The synthesis and characterization of ligands **A** and **B**, as well as complexes **1** and **2**, are described in our previous report.[1] X-ray crystallographic data for homocomplexes **1** (CCDC # 951758) and **2** (CCDC # 951759) can be found in our previous report.[1] The minimized structures of cages **3-5** were obtained via density functional calculations, and were optimized using the dispersion-corrected B97-D density functional[2,3] in the 6-31G(d) basis set.[4]

Synthesis of Compounds

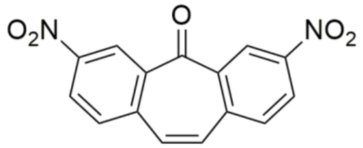

3,7-Dinitrodibenzosuberenone (*S-1*):

3,7-Dinitrodibenzosuberone (100 mg, 0.33 mmol) was added to a 50 mL round bottom flask with stir bar, followed by the addition of benzene (25 mL). 1.1 equivalents N-bromosuccinimide (64.8 mg, 0.36 mmol) was slowly added to the flask, followed by 0.1 eq benzoyl peroxide (8.0 mg, 0.03 mmol). The reaction mixture was refluxed for 12 h, after which the solid was filtered and dried. The crude solid was placed into a 50mL round bottom flask followed by acetone (25 mL). Potassium iodide (60.4 mg, 0.33mmol) was added to the flask and the mixture was stirred at room temperature for 4 h. The reaction mixture was then filtered and the product collected as a light yellow solid (47 mg, 50 %). ^{1}H NMR (400 MHz, DMSO-d_6): δ 8.70.25 (d, J = 2.5 Hz, 2H), 8.53 (dd, J = 8.5, 2.6 Hz, 2H), 7.99 (d, J = 8.5 Hz, 2H),

6.58 (s, 2H). ^{13}C NMR (150 MHz, DMSO-d_6): δ 180.2, 148.4, 140.0, 138.4, 134.6, 134.1 127.7, 126.3. HRMS (ESI) m/z calcd for $C_{15}H_8N_2O_5$ (**S-1**$^+$) 296.0418, found 296.0228.

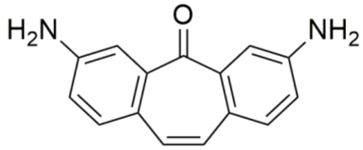

3,7-Diaminodibenzosuberenone (C):

S-1 (400 mg, 1.34 mmol) was added to a 50 mL round bottom flask with stir bar, followed by addition of Raney© 2800 Ni suspension in water (1.0 mL) and MeOH (25 mL). The flask was fixed with a septum and purged with nitrogen gas. Hydrazine monohydrate (2.0 mL, 41.2 mmol) was slowly added. After the addition, the reaction was stirred at room temperature. After 24 h the reaction mixture was diluted with acetone (100 mL) followed by filtering through celite. After evaporating the solvent *in vacuo*, the residue was triturated in deionized water (200 mL) before being filtered using celite. The filter was rinsed clean using MeOH (150 mL) before evaporating the solvent *in vacuo* to give an orange-yellow solid. This was recrystallized from EtOH to give product as an orange solid (162 mg, 50 %). ^{1}H NMR (400 MHz; DMSO-d_6) δ 7.33 (s, 2H), 7.31 (d, J = 5.9 Hz, 2H), 6.89 (dd, J = 5.9, 2.2 Hz, 2H), 6.69 (d, J = 2.2 Hz, 2H). ^{13}C NMR (150 MHz, DMSO-d_6): δ 191.4, 149.8, 138.7, 133.4, 127.6, 126.0, 119.7, 113.3. HRMS (ESI) m/z calcd. for $C_{15}H_{13}N_2O$ ([**C**•H]$^+$) 237.1178, found 237.1248.

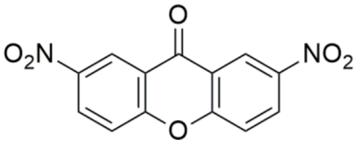

2,7-Dinitroxanthone (*S-2*)

Fuming nitric acid (15 mL) and concentrated sulfuric acid (10 mL) were added to a 250 mL round bottom flask and chilled to 0 °C. Xanthone (2 g, 10.1 mmol) was slowly added to the solution in 200 mg portions with vigorous stirring over a period of 30 minutes. The yellow solution was allowed to stir for an additional hour at 0 °C. The mixture was then poured into a beaker containing 150 g ice with vigorous stirring and the precipitate was filtered. The pale yellow crude product was then recrystallized from nitromethane to yield an off white solid (1.98 g 68 %) ^{1}H NMR (400 MHz, DMSO-d_6): δ 9.25 (d, J = 2.8 Hz, 2H), 8.65 (dd, J = 9.2, 2.8 Hz 2H), 7.75 (d, J = 9.2 Hz, 2H). ^{13}C NMR (100 MHz, DMSO-d_6): δ 175.6, 159.5, 144.8, 131.0, 122.7, 121.8, 121.5. HRMS (ESI) m/z calcd. for $C_{13}H_6N_2O_6$ (**S-2**$^+$) 286.0257, found 286.0228.

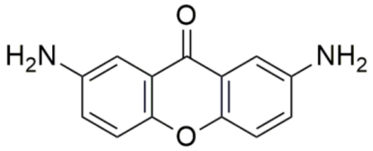

2,7-Diaminoxanthone (D)

S-2 (100 mg, 0.33 mmol) was placed in a 50 mL round bottom flask with a stir bar followed by 10 mL of concentrated hydrochloric acid. Then, 4.5 equivalents of tin(II)chloride dihydrate was added to the flask. The reaction was refluxed for 12 h with stirring, then allowed to cool to room temperature. The reaction mixture was diluted with 50mL deionized water and brought to a pH of 8.5 using 2 M NaOH. The yellow solution was then extracted using ethyl acetate until no color was present in the aqueous layer (3 x 15 mL). The solution was dried using anhydrous MgSO4, filtered and the solvent removed in vacou to yield an orange solid (57 mg, 72 %). ^{1}H NMR (400 MHz, DMSO-d_6): δ 7.33 (d, J = 8.8 Hz, 2H), 7.22 (d, J = 2.8 Hz, 2H), 7.07 (dd, J = 9.0 Hz, 2H), 5.32 (s, 4H). ^{13}CNMR (100 MHz, DMSO-d_6): δ 176.8, 148.7, 145.1, 123.9, 122.1, 119.2, 106.8. HRMS (ESI) m/z calcd. for $C_{13}H_{11}N_2O_2$ ([**D**•H]$^+$) 227.0713, found 227.0628.

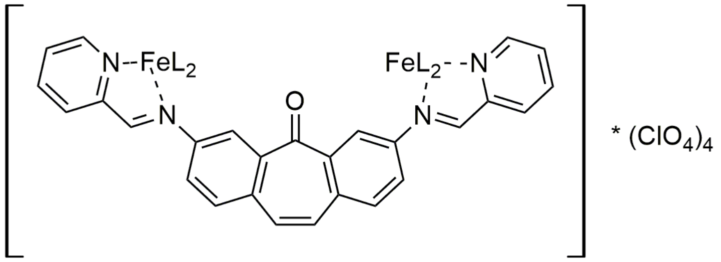

Cage 3:

Dianiline **C** (38.3 mg, 0.161 mmol), 2-pyridine carboxaldehyde (27.0 μL, 0.32 mmol) and $Fe(ClO_4)_2$•xH_2O (35.1 mg) were combined in anhydrous MeCN (5 mL) in a 50 mL round-bottomed flask under a blanket of N_2 gas. The solution was then heated at 45 °C for 10 h with stirring. The purple solution was diluted with Et_2O (25 mL), cooled to -25 °C followed by filtration of the resulting precipitate. After drying, the product was isolated as a purple solid (65.0 mg, 92 %) ^{1}H NMR (400 MHz; CD_3CN) δ 8.74 (s, 2H), 8.50 (d, J = 7.4 Hz, 2H), 8.44 (t, J = 7.3 Hz, 2H), 7.8 (t, J = 5.2 Hz, 2H), 7.56 (d, J = 8.3 Hz, 2H), 7.43 (d, J = 5.3 Hz, 2H), 7.2 (s, 2H), 6.56 (d, J = 2.2 Hz, 2H), 5.62 (dd, J = 8.2, 2.2 Hz, 2H). ^{13}C NMR (150 MHz, DMSO-d_6): δ 186.4, 176.4, 158.6, 156.4, 150.4, 140.4, 138.5, 135.8, 134.8, 132.7, 131.8, 130.4, 125.5, 122.9. HRMS (ESI) m/z calcd. for $C_{81}H_{60}Fe_2N_{12}O_3$ ([**3**]$^{4+}$) 339.0897, found 339.0802

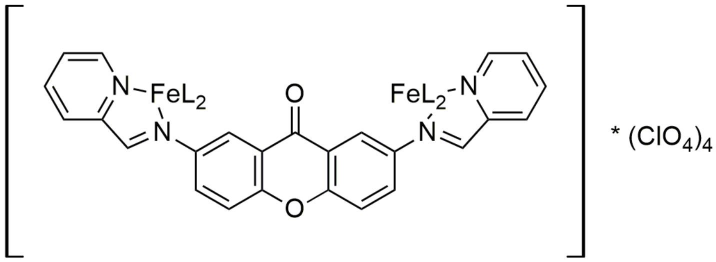

Cage 4:

Dianiline **D** (100 mg, 0.44 mmol), 2-pyridine carboxaldehyde (107 µL, 0.88 mmol) and $Fe(ClO_4)_2$•xH_2O (96.3 mg) were combined in anhydrous MeCN (10 mL) in a 50 mL round-bottomed flask under a blanket of N_2, followed by heating to 45 °C for 10 h. The solution was then cooled to room temperature, diluted with Et_2O (30 mL), and cooled to -25 °C followed by filtration of the resulting precipitate. Drying product *in vacuo* gave product as a purple solid (190 mg, 96 %). ^1H NMR (400 MHz; CD_3CN) δ 11.13 (m, 2H), 9.31 (s, 2H), 8.98 (m, 2H), 8.48 (s, 2H), 8.34 (s, 2H), 7.33 (m, 2H), 6.39 (s, 2H), 5.44 (d, J = 8.5 Hz, 2H). Cage **4** was not soluble enough to collect a ^{13}C NMR spectrum within a reasonable amount of time. HRMS (ESI) m/z calcd for $C_{75}H_{54}Fe_2N_{12}O_{14}Cl_2$ ([**4**•$(ClO_4)_2]^{2+}$) 761.7364, found 761.9462.

NMR Spectral Data

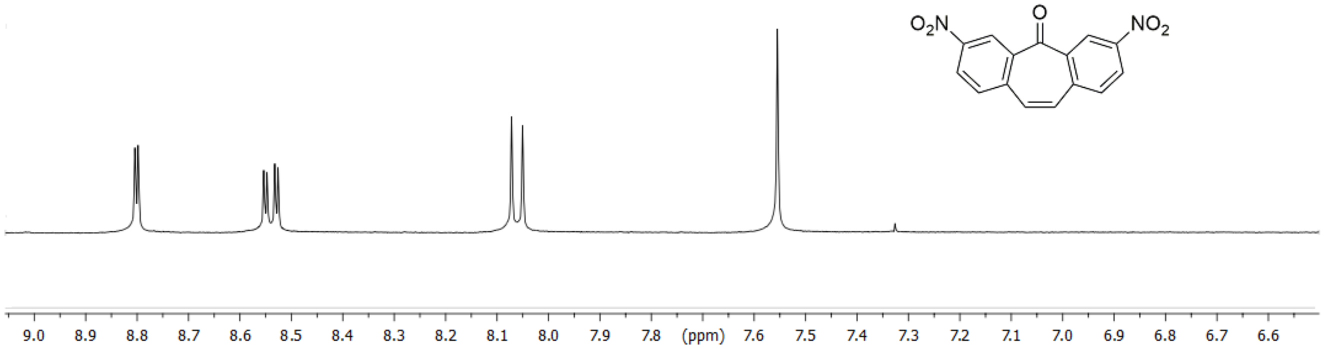

Figure S1: ^1H NMR spectrum of ***S-1*** (DMSO, 400 MHz, 298 K).

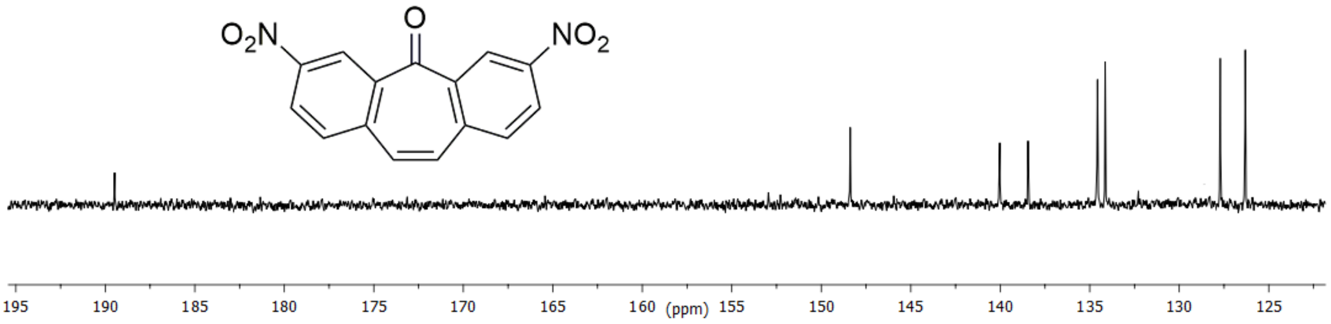

Figure S2: ^{13}C NMR spectrum of ***S-1*** (DMSO, 150 MHz, 298 K).

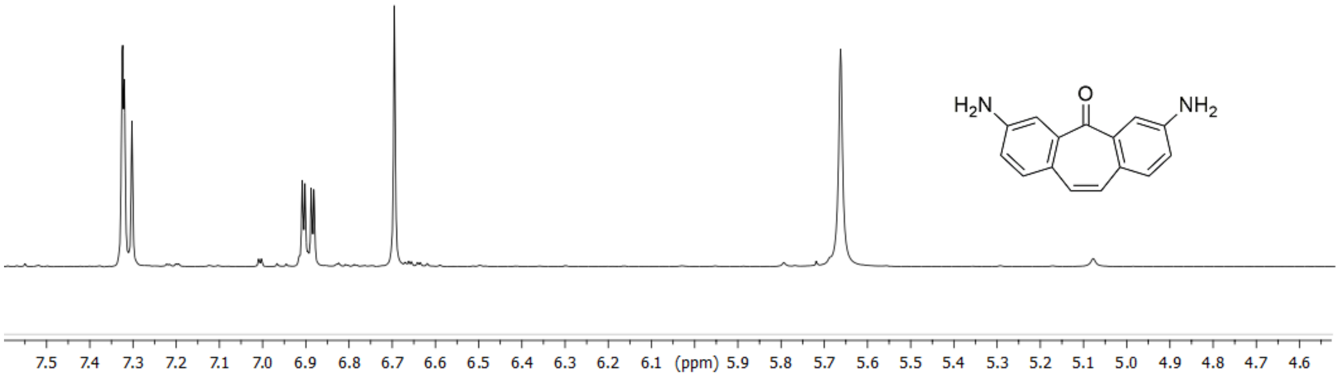

Figure S3: ^{1}H NMR spectrum of **C** (DMSO, 400 MHz, 298 K).

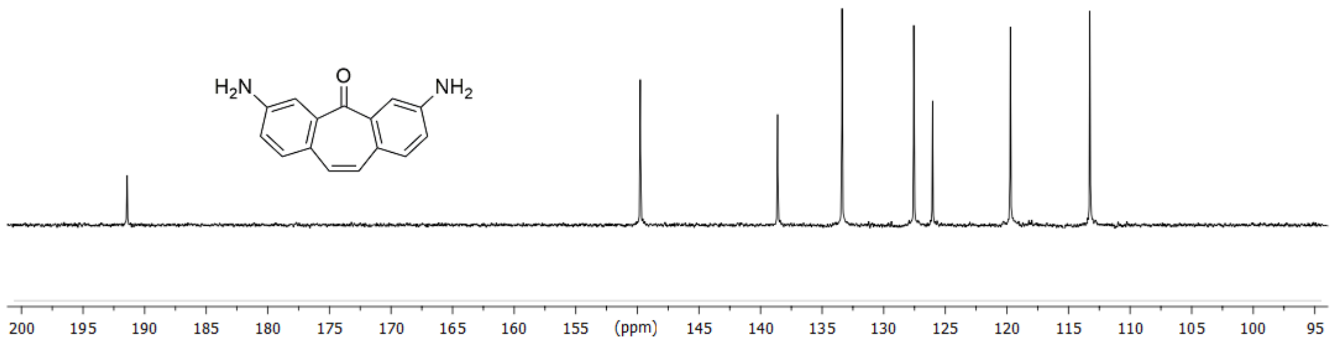

Figure S4. ^{13}C NMR spectrum of **C** (DMSO, 150 MHz, 298 K).

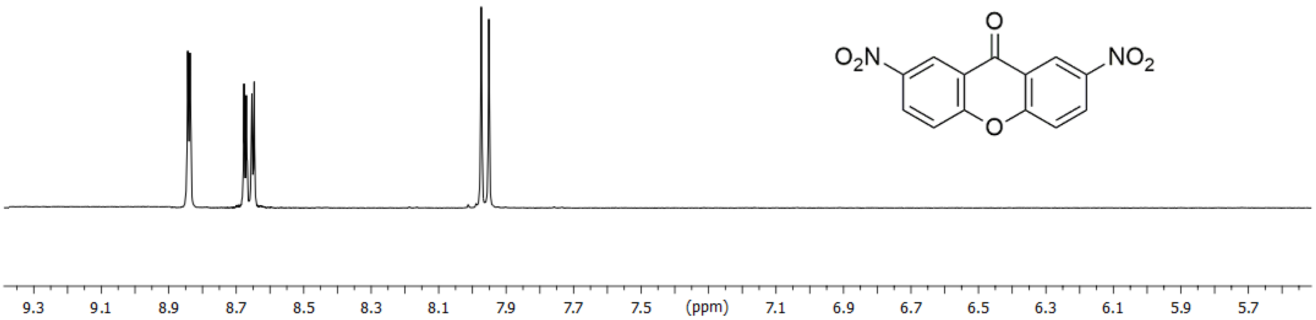

Figure S5: ^{1}H NMR spectrum of ***S-2*** (DMSO, 400 MHz, 298 K).

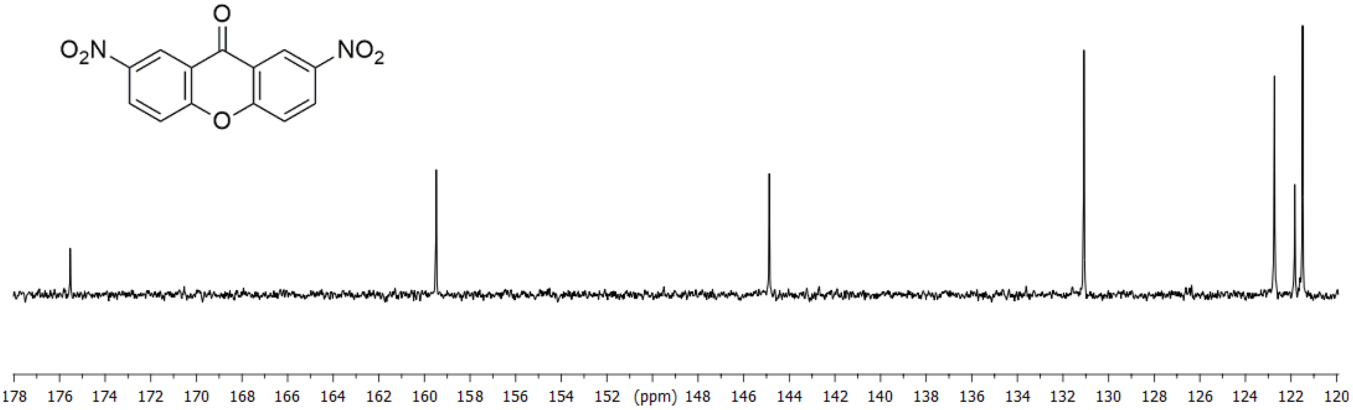

Figure S6: ^{13}C NMR spectrum of ***S-2*** (DMSO, 150 MHz, 298 K).

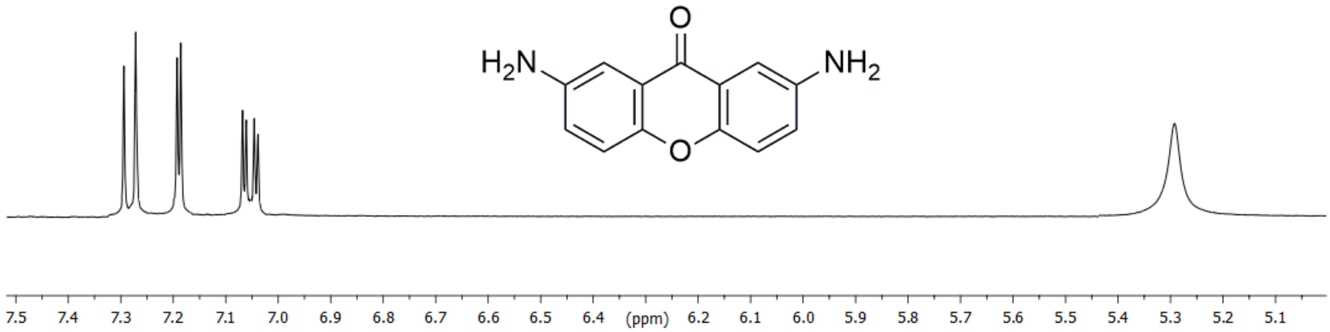

Figure S7:. ^{1}H NMR spectrum of **D** (DMSO, 400 MHz, 298 K).

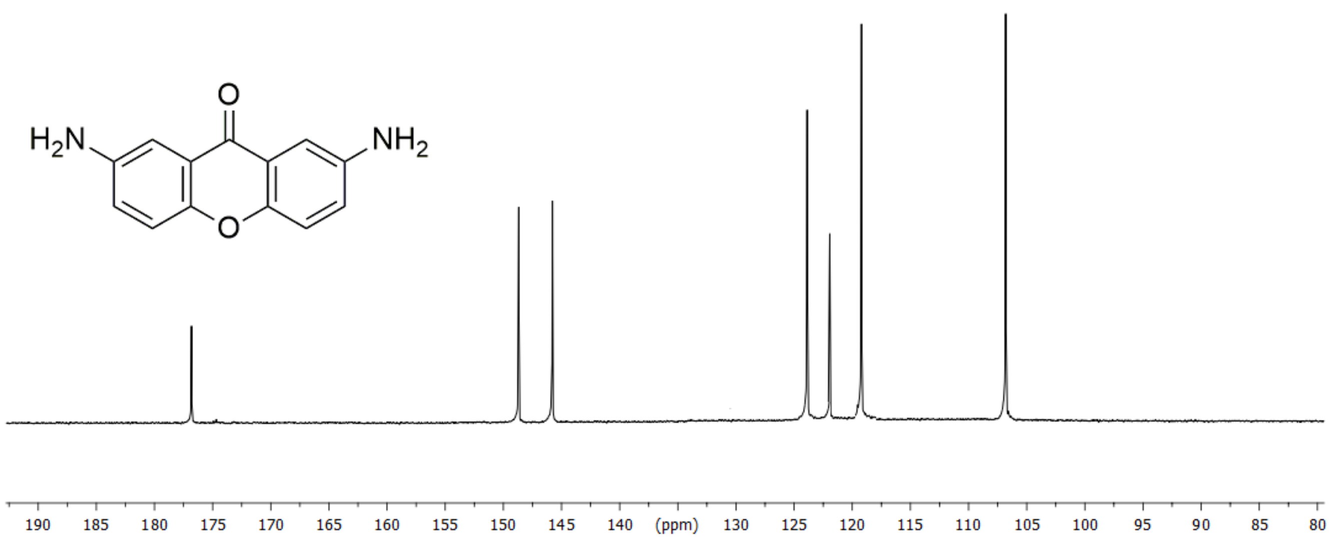

Figure S8: ^{13}C NMR spectrum of **D** (DMSO, 150 MHz, 298 K).

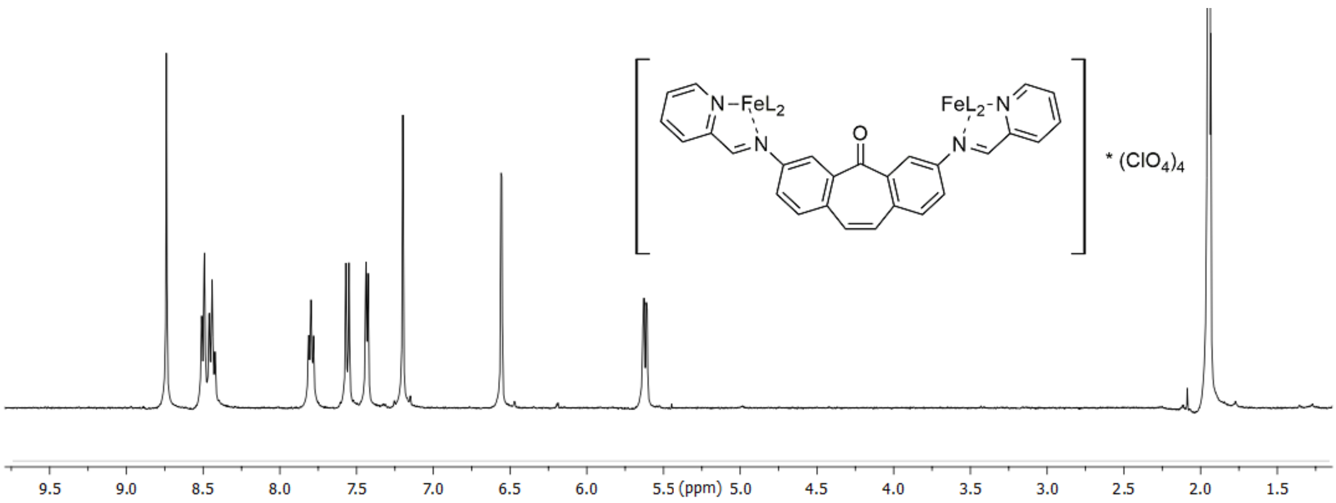

Figure S9: ^{1}H NMR spectrum of Cage **3** (CD_3CN, 400 MHz, 298 K).

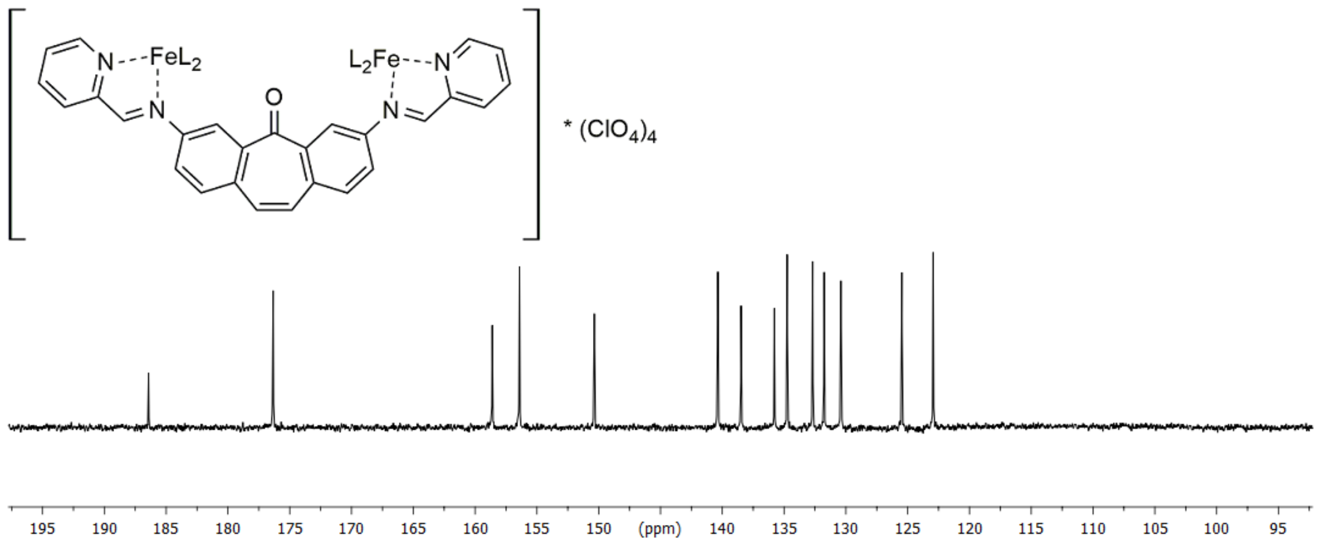

Figure S10: ^{13}C NMR spectrum of Cage **3** (CD_3CN, 150 MHz, 298 K).

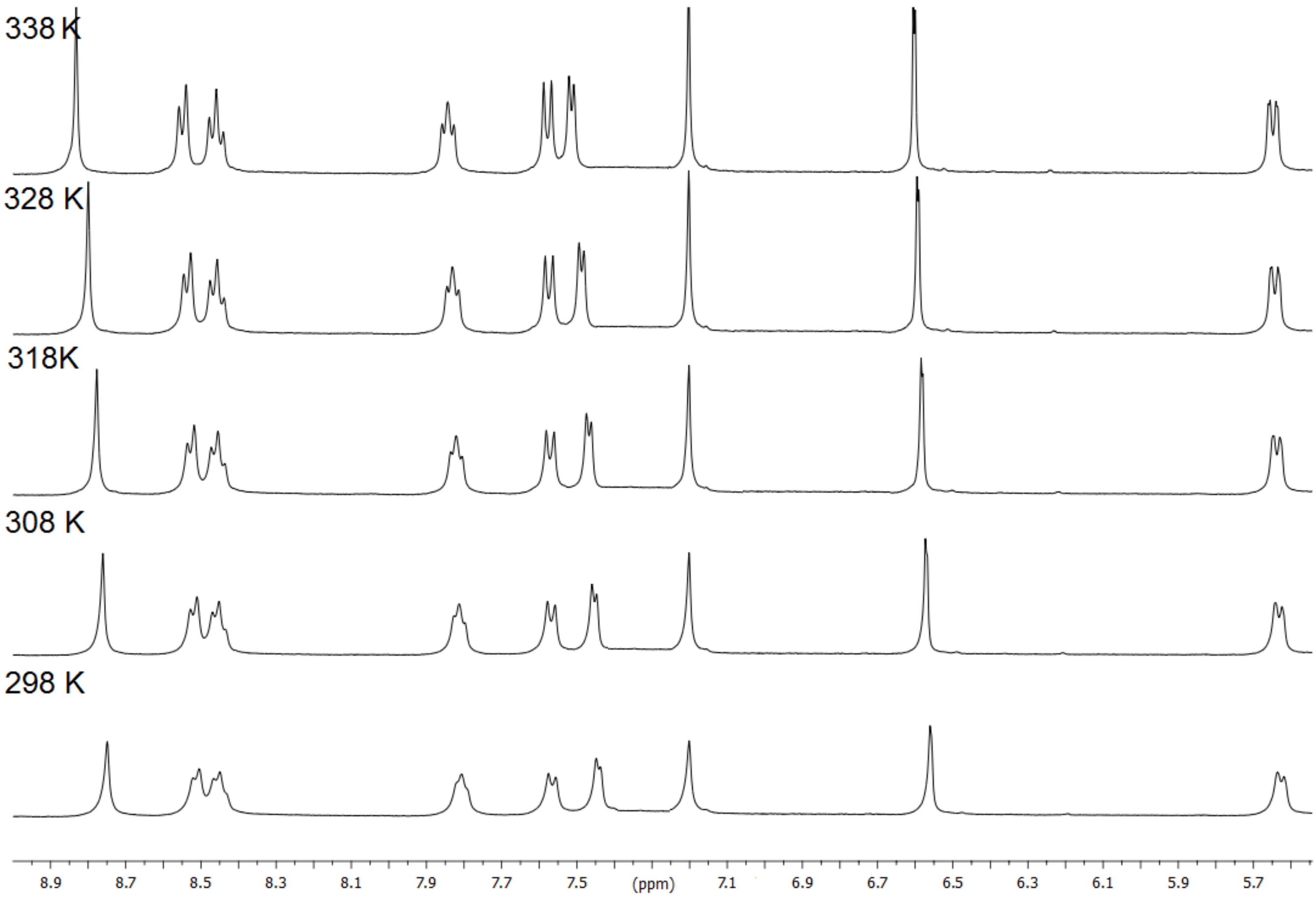

Figure S11: ^{1}H NMR scans of Cage **3** at various temperatures (CD_3CN, 600 MHz, 298-338 K).

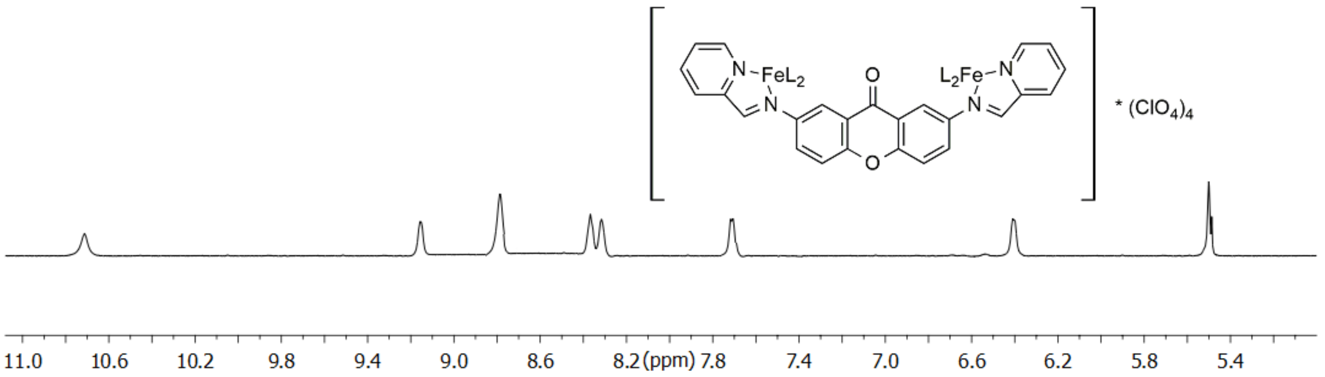

Figure S12: ^{1}H NMR spectrum of Cage **4** (CD_3CN, 400 MHz, 298 K).

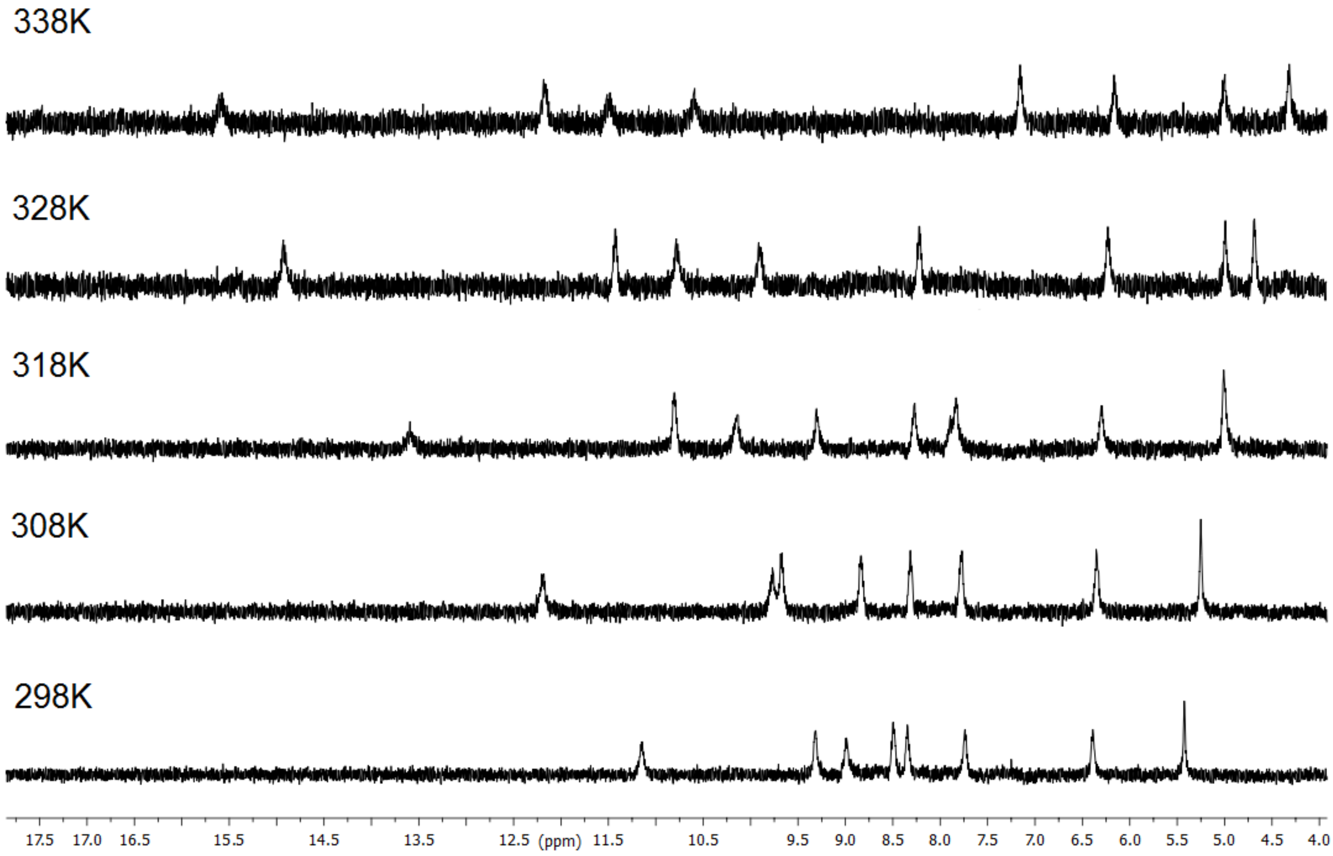

Figure S13: ^{1}H NMR scans of Cage **4** at various temperatures (CD_3CN, 600 MHz, 298K - 338K).

Mass Spectral Data

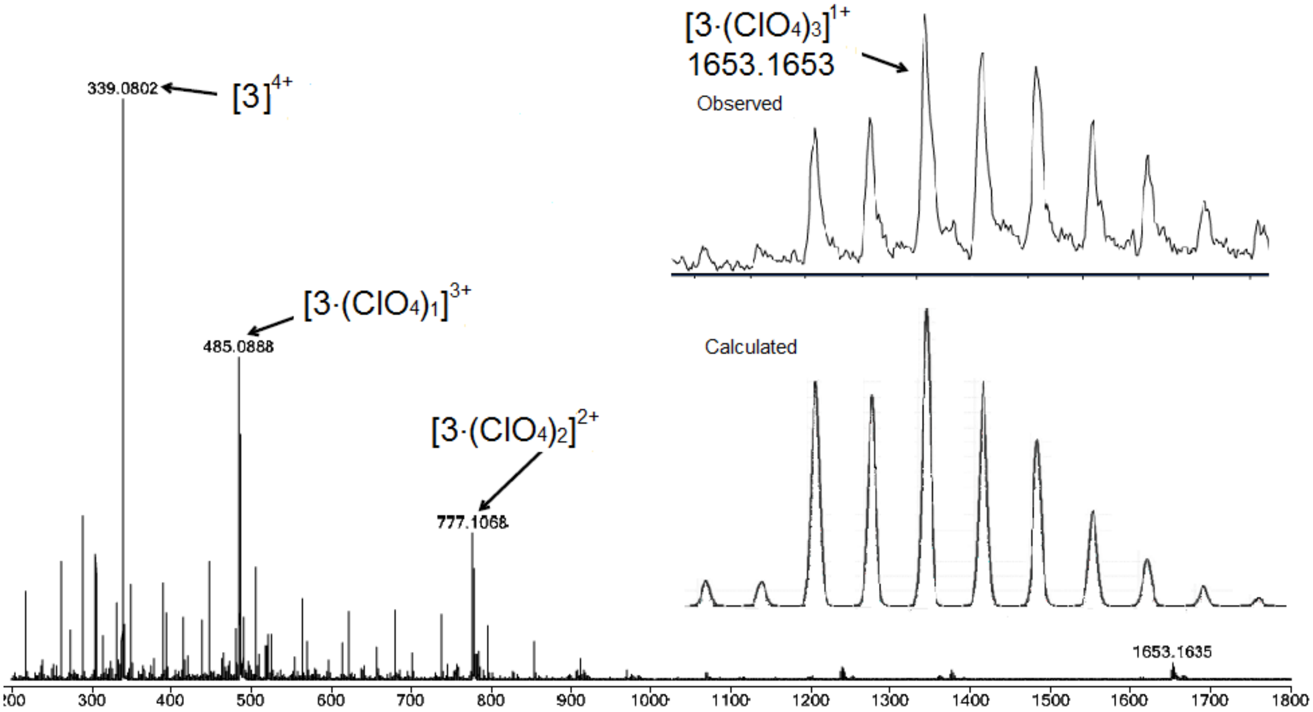

Figure S14: ESI-MS of Cage **3** (CH_3CN).

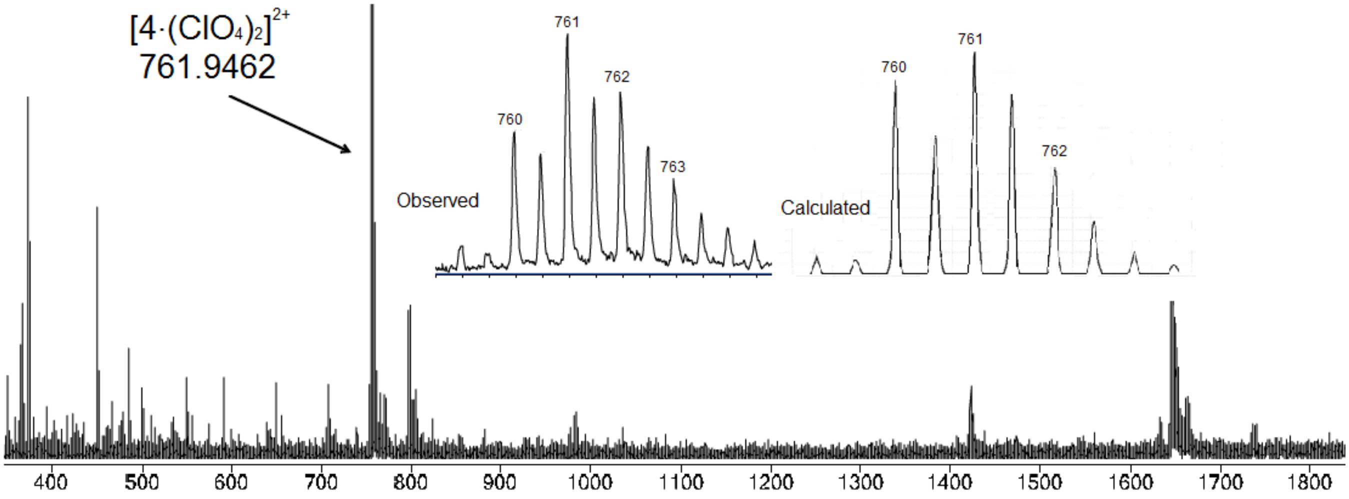

Figure S15: ESI-MS of Cage **4** (CH_3CN).

Assembly Mixing Experiments

General mixing procedure: All mixing experiments were performed in an NMR tube. One equivalent of dianiline **A** (3.5 mg, 0.015 mmol) and one equivalent of dianiline **B** (3.5 mg 0.015 mmol) were placed in an NMR tube. Deuterated acetonitrile (400 µL) was added to the tube and a proton spectrum of the dianiline mixture obtained. 2 equivalents of 2-formylpyridine were added (5µL, 0.029 mmol) followed by 0.66 equivalents of iron perchlorate (100 µL of 0.098 M $Fe(ClO_4)_2$•xH_2O in CD_3CN). A spectrum of the mixture was obtained. The tube was heated at 80 °C for 8 h. Another spectrum was taken after heating to show the favored cage and the unfavored dianiline ligand. A second 2 eq. of 2-formylpyridine and 0.66 eq. of iron perchlorate were added to the tube and a proton spectrum obtained. The tube was heated to 80 °C for 8 h. A final spectrum was obtained after heating to show both cages in solution.

Mixing Experiment Between Ligands A and C

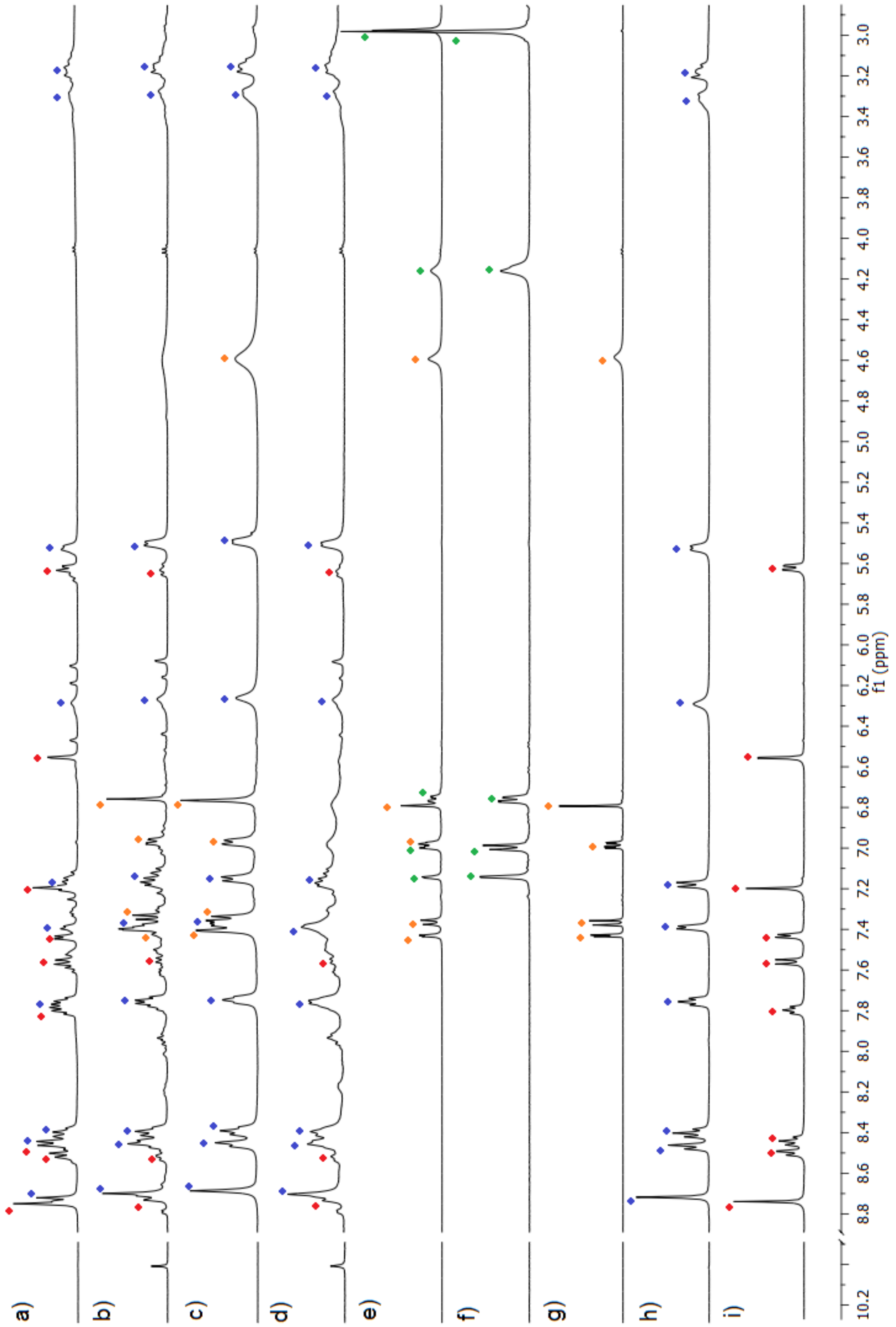

Figure S16: ^{1}H NMR spectra of mixing experiment between ligands **A** and **C** (CD_3CN, 400 MHz, 298K). i) Cage **3** (red), h) Cage **1** (blue), g) Dianiline **C** (orange), f) Dianiline **A** (green), e) Dianiline mixture, d) Mixture with first addition of 2-formylpyridine and iron with no heat, c) Mixture with first addition after 8 h at 80 °C, b) Mixture after second addition of 2-formylpyridine and iron with no heat, a) mixture after second addition and 8 h heat at 80 °C.

Mixing Experiment Between Ligands A and B

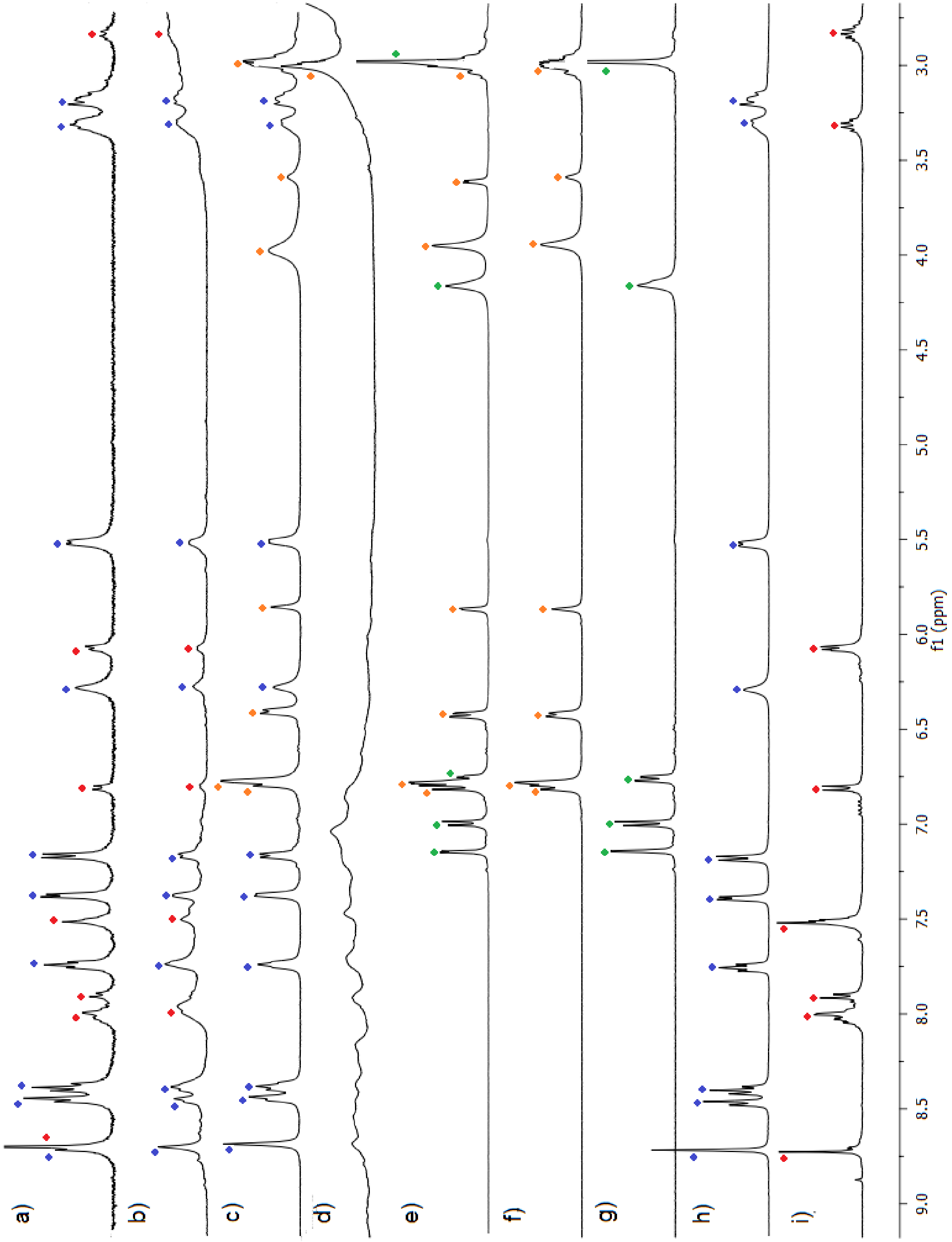

Figure S17: ^{1}H NMR spectra of mixing experiment between ligands **A** and **B** (CD_3CN, 400 MHz, 298K). i) Cage **2** (red), h) Cage **1** (blue), g) Dianiline **A** (green), f) Dianiline **B** (orange), e) Dianiline mixture, d) Mixture with first addition of 2-formylpyridine and iron with no heat, c) Mixture with first addition after 8 h at 80 °C, b) Mixture after second addition of 2-formylpyridine and iron with no heat, a) mixture after second addition and 8 h heat at 80 °C.

Mixing Experiment Between Ligands B and C

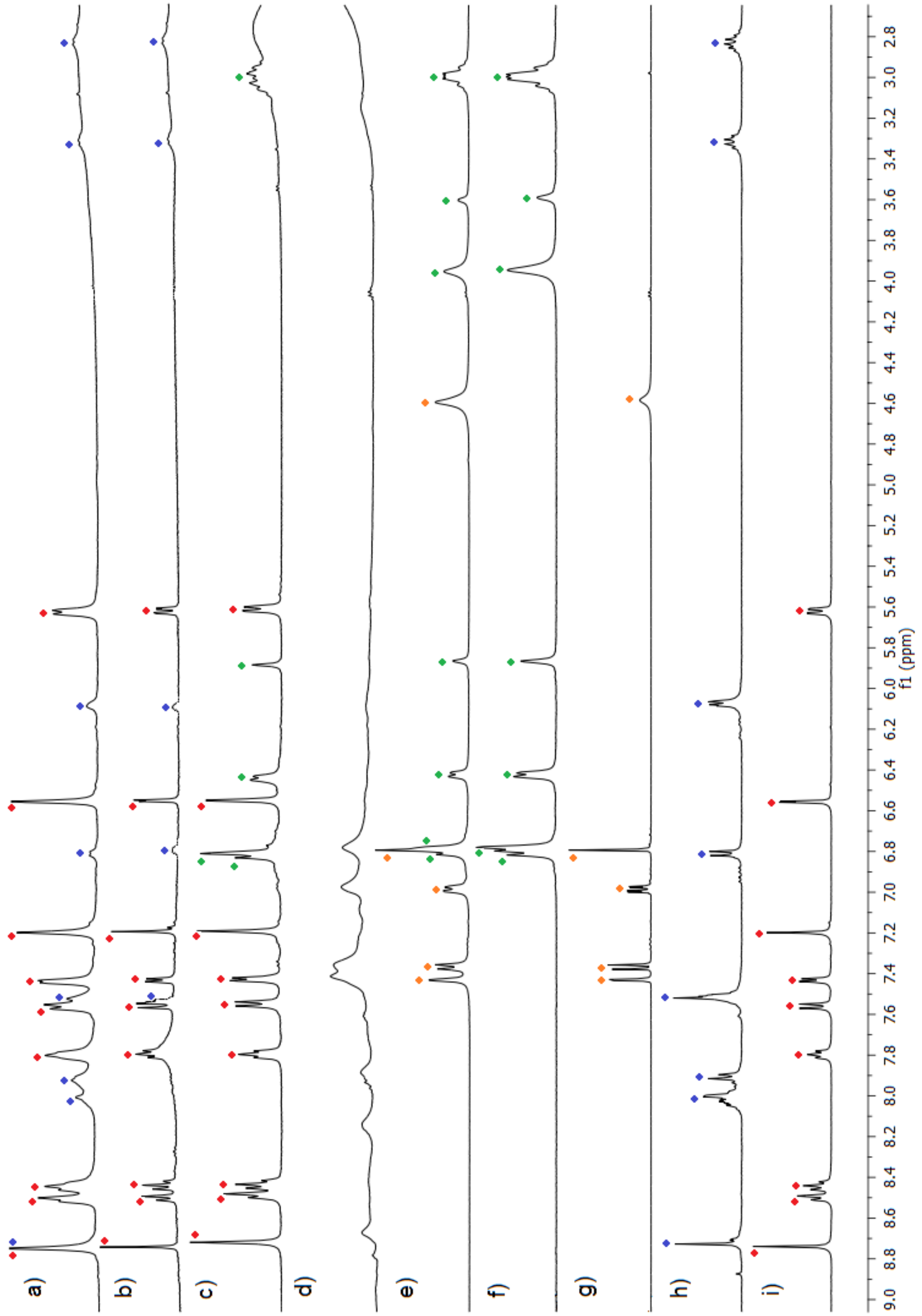

Figure S18: ^{1}H NMR spectra of mixing experiment between ligands **B** and **C** (CD_3CN, 400 MHz, 298K). i) Cage **3** (red), h) Cage **2** (blue), g) Dianiline **C** (orange), f) Dianiline **B** (green), e) Dianiline mixture, d) Mixture with first addition of 2-formylpyridine and iron with no heat, c) Mixture with first addition after 8 h at 80 °C, b) Mixture after second addition of 2-formylpyridine and iron with no heat, a) mixture after second addition and 8 h heat at 80 °C.

Mixing Experiment Between Ligands A and D

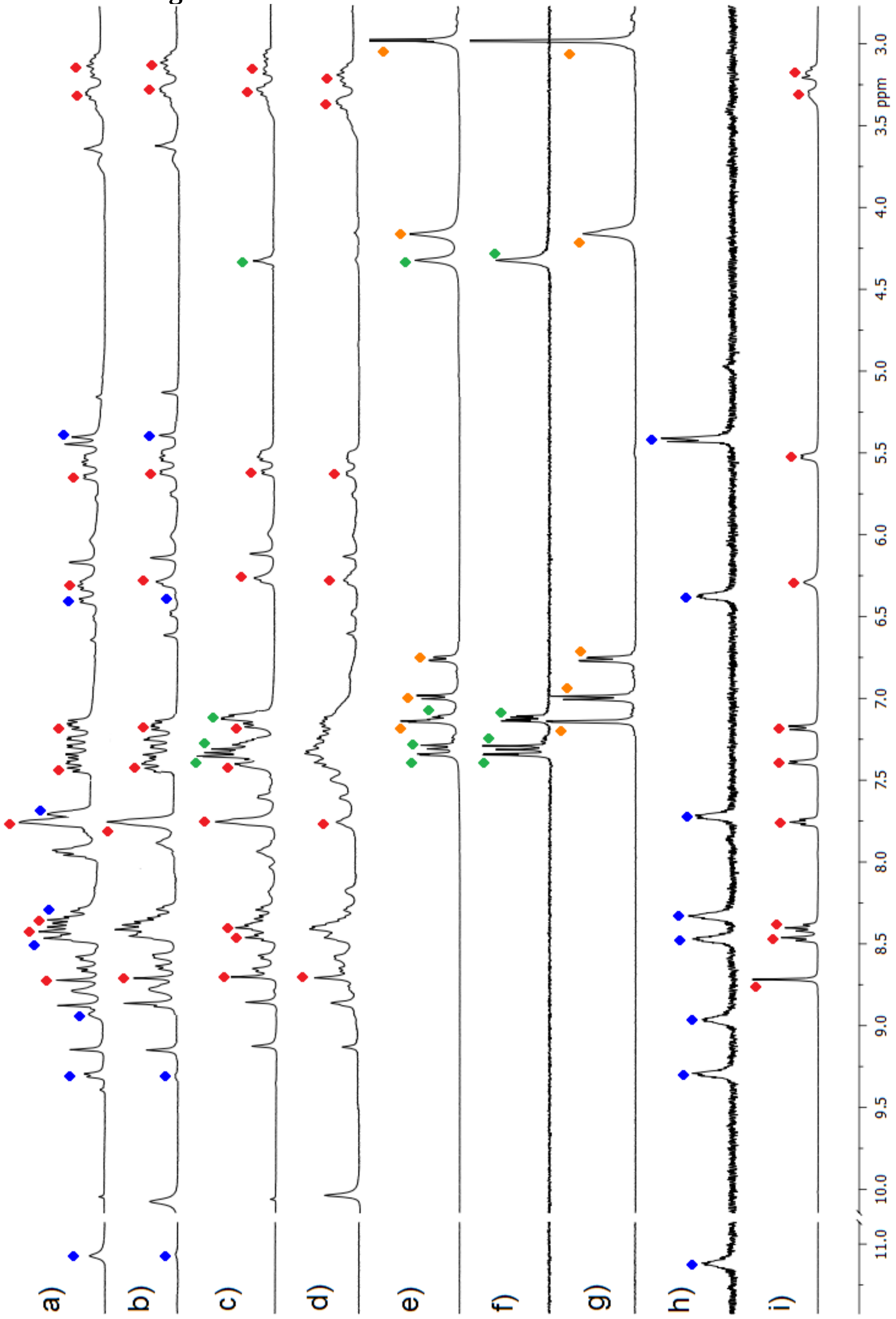

Figure S19: ^{1}H NMR spectra of mixing experiment between ligands **A** and **D** (CD_3CN, 400 MHz, 298K). i) Cage **1** (red), h) Cage **4** (blue), g) Dianiline **A** (orange), f) Dianiline **D** (green), e) Dianiline mixture, d) Mixture with first addition of 2-formylpyridine and iron with no heat, c) Mixture with first addition after 8 h at 80 °C, b) Mixture after second addition of 2-formylpyridine and iron with no heat, a) mixture after second addition and 8 h heat at 80 °C.

Mixing Experiment Between Ligands C and D

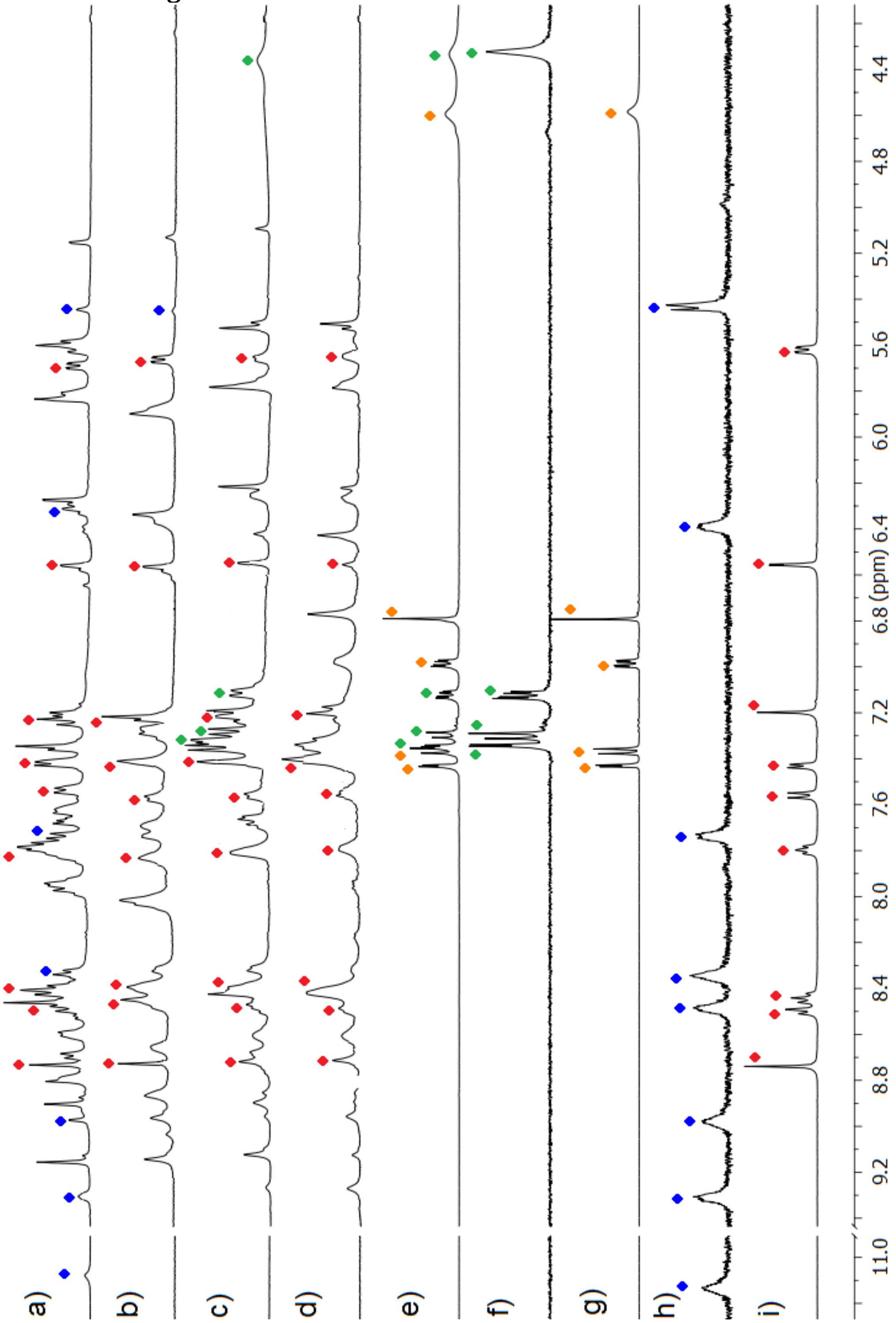

Figure S20: ^{1}H NMR spectra of mixing experiment between ligands **C** and **D** (CD_3CN, 400 MHz, 298K). i) Cage **3** (red), h) Cage **4** (blue), g) Dianiline **C** (orange), f) Dianiline **D** (green), e) Dianiline mixture, d) Mixture with first addition of 2-formylpyridine and iron with no heat, c) Mixture with first addition after 8 h at 80 °C, b) Mixture after second addition of 2-formylpyridine and iron with no heat, a) mixture after second addition and 8 h heat at 80 °C.

Mixing Experiment Between Ligands B and D

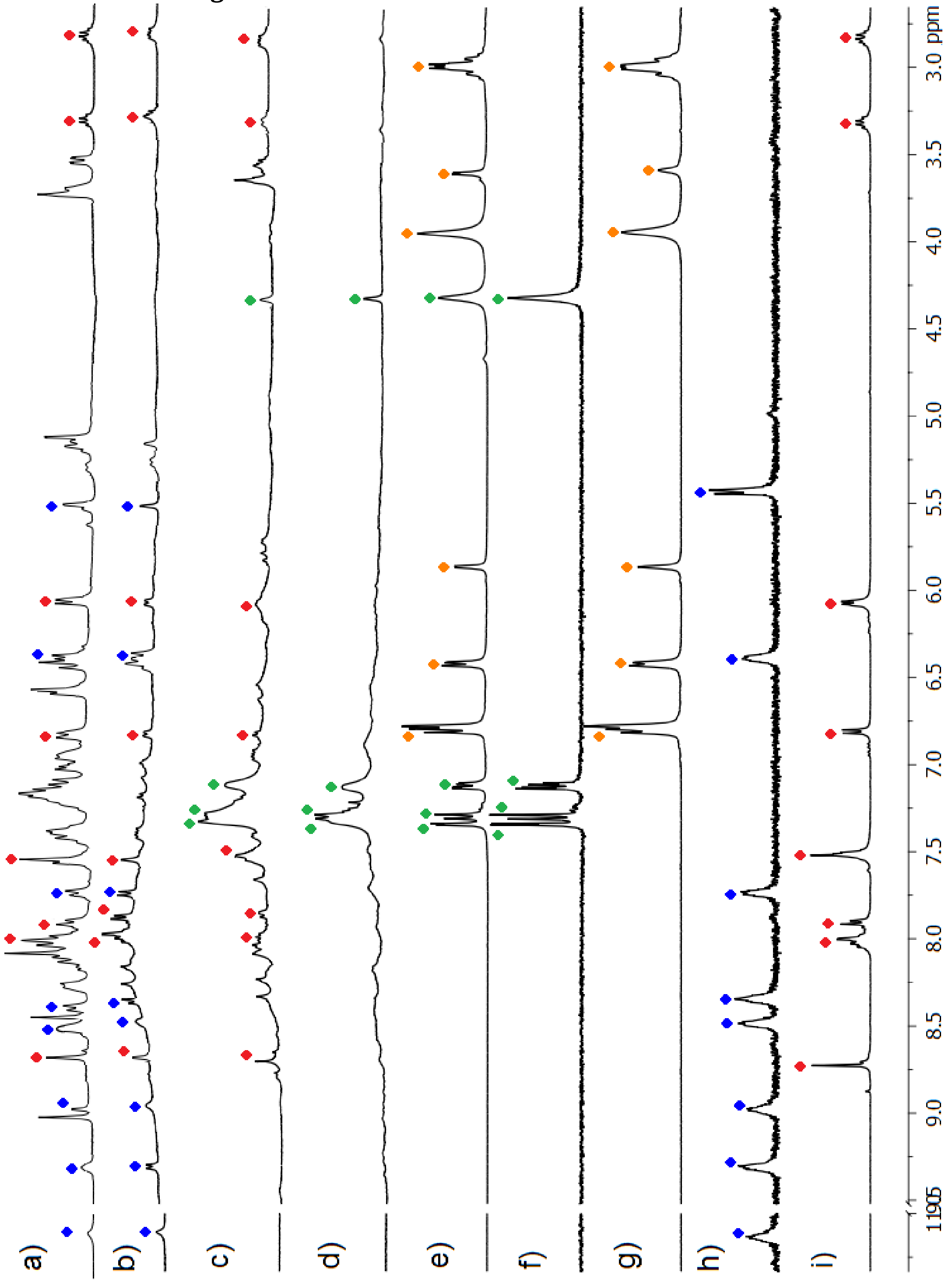

Figure S21: ^{1}H NMR spectra of mixing experiment between ligands **B** and **D** (CD_3CN, 400 MHz, 298K). i) Cage **2** (red), h) Cage **4** (blue), g) Dianiline **B** (orange), f) Dianiline **D** (green), e) Dianiline mixture, d) Mixture with first addition of 2-formylpyridine and iron with no heat, c) Mixture with first addition after 8 h at 80 °C, b) Mixture after second addition of 2-formylpyridine and iron with no heat, a) mixture after second addition and 8 h heat at 80 °C.

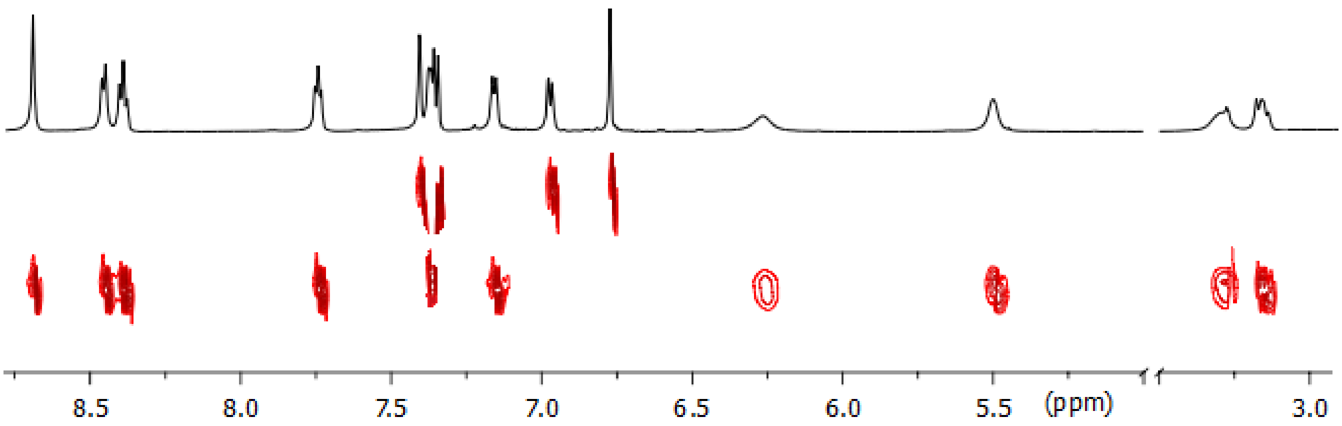

Figure S22: ^{1}H-DOSY NMR spectrum of mixing between dianilines **A** and **C** after 1 addition of $Fe(ClO_4)_2$ and 2-formylpyridine and 8 h heat at 80°C (CD_3CN, 600 MHz, 298 K, Δ = 100 ms, δ = 2.6 µs, Diffusion Coefficient = 8.39 x10^{-10} m^2/s for cage **1** vs. 2.15 x10^{-9} m^2/s for dianiline **C** vs. 4.92 x10^{-9} m^2/s for solvent).

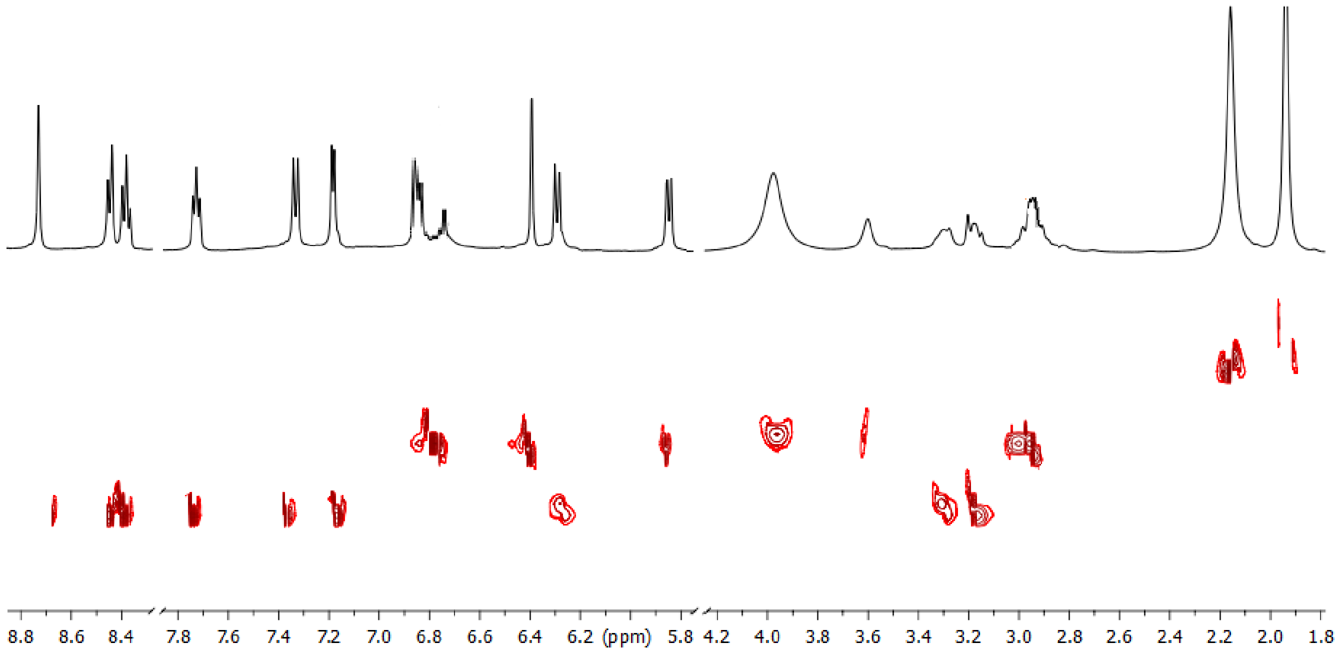

Figure S23: ^{1}H-DOSY NMR spectrum of mixing between dianilines **A** and **B** after 1 addition of $Fe(ClO_4)_2$ and 2-formylpyridine and 8 h heat at 80°C (CD_3CN, 600 MHz, 298 K, Δ = 100 ms, δ = 2.6 µs, Diffusion Coefficient = 7.21 x10^{-10} m^2/s for cage **1** vs. 1.91 x10^{-9} m^2/s for dianiline **B** vs. 4.16 x10^{-9} m^2/s for solvent).

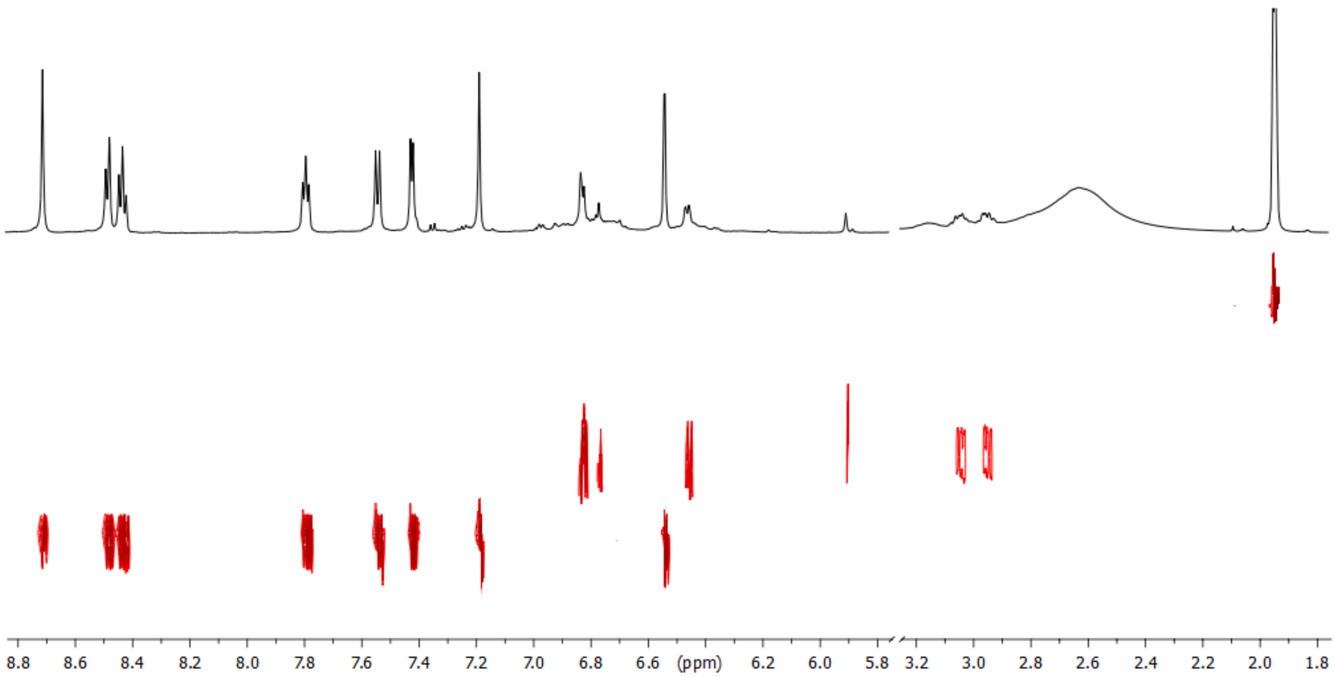

Figure S24: ^{1}H-DOSY NMR spectrum of mixing between dianilines **B** and **C** after 1 addition of $Fe(ClO_4)_2$ and 2-formylpyridine and 8 h heat at 80°C (CD_3CN, 600 MHz, 298 K, Δ = 100 ms, δ = 2.6 μs, Diffusion Coefficient = 9.23 $x10^{-10}$ m^2/s for cage **3** vs. 1.66 $x10^{-9}$ m^2/s for dianiline **B** vs. 5.57 $x10^{-9}$ m^2/s for solvent).

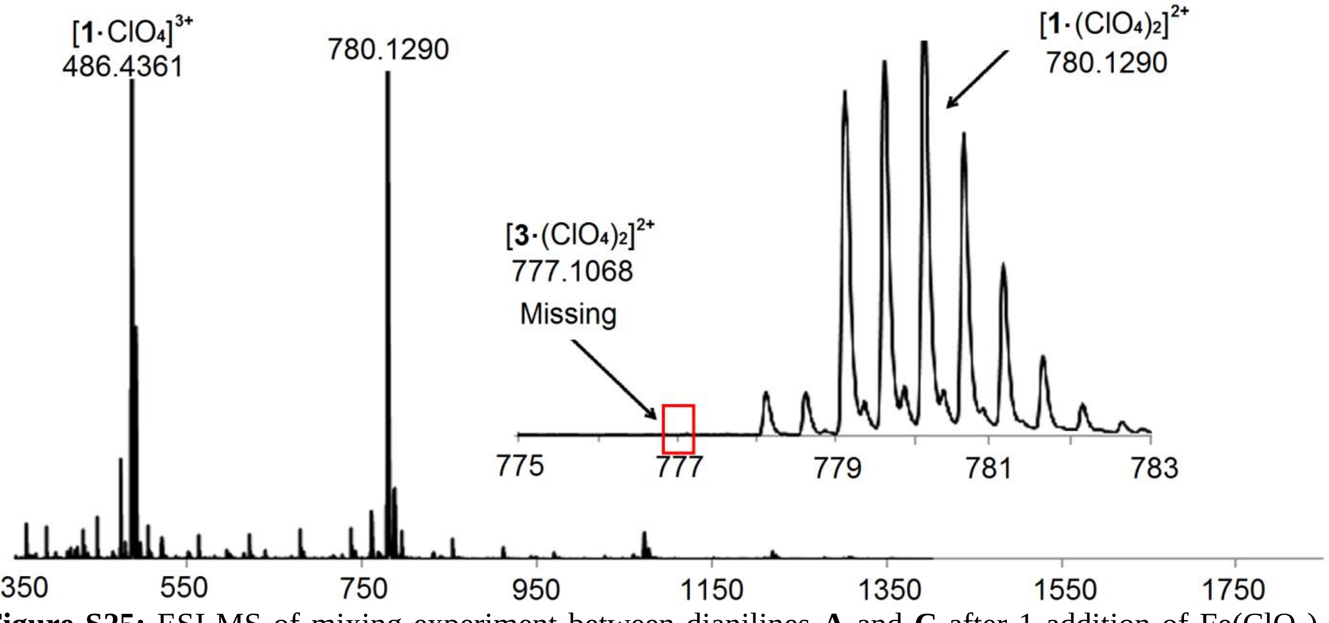

Figure S25: ESI-MS of mixing experiment between dianilines **A** and **C** after 1 addition of $Fe(ClO_4)_2$ and 2-formylpyridine and 8 h heat at 80°C. Only Cage **1** and Ligand **C** were detected.

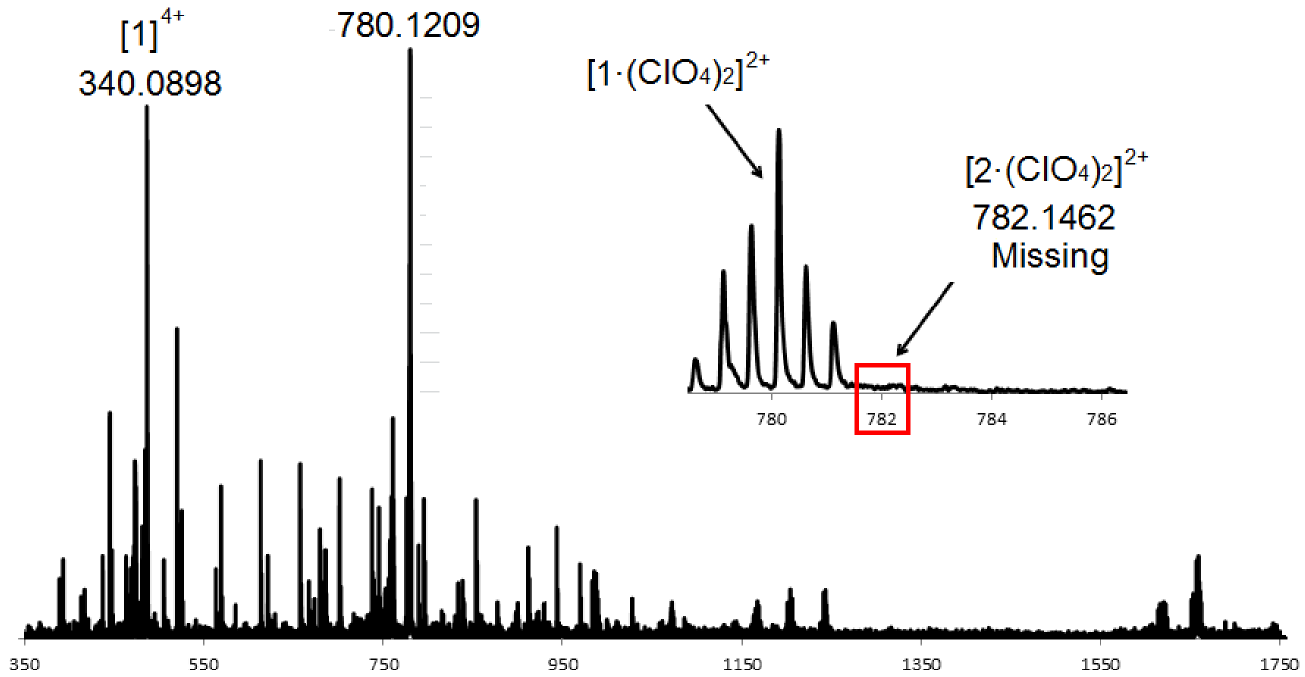

Figure S26: ESI-MS of mixing experiment between dianilines **A** and **B** after 1 addition of $Fe(ClO_4)_2$ and 2-formylpyridine and 8h heat at 80°C. Only Cage **1** and ligand **B**

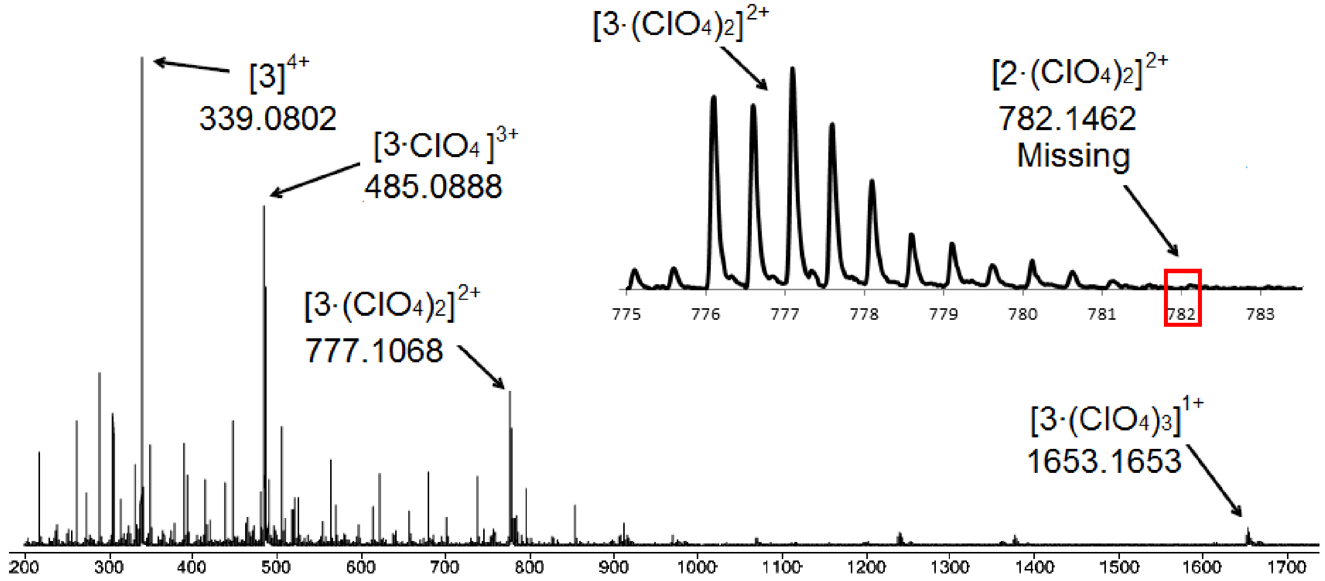

Figure S27: ESI-MS of mixing experiment between dianilines **B** and **C** after 1 addition of $Fe(ClO_4)_2$ and 2-formylpyridine and 8 h heat at 80°C. Only Cage **3** and ligand **B** were detected.

Ligand Displacement Experiments

General displacement procedure: All displacement experiments were performed in an NMR tube. One equivalent of preformed Cage **1** (8.6 mg, 0.005 mmol) and three equivalents of dianiline **B** (3.5 mg 0.015 mmol) were placed in an NMR tube. Dry deuterated acetonitrile (400 µL) was added to the tube and a proton spectrum of the starting mixture obtained. The tube was heated at 80°C for 8 h. A second spectrum was obtained after heating to verify whether the preformed cage was displaced by the free dianiline ligand. Experiments were repeated with the addition of 6 molar equivalents of water and heated at 55°C for 1 hour.

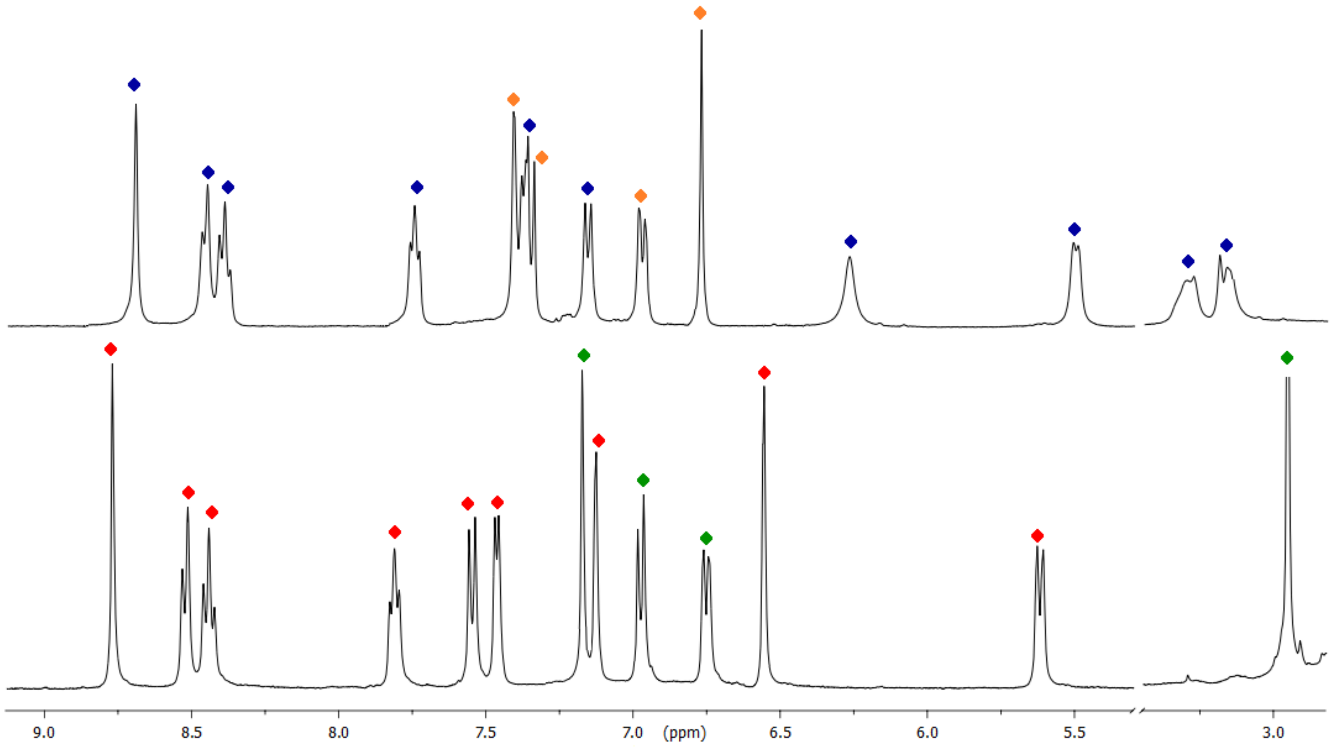

Figure S28: ^{1}H NMR spectra of anhydrous displacement experiment between cage **3** and dianiline **A** (CD_3CN, 400 MHz, 298K). Top: Cage **1** (blue) and displaced dianiline **C** (orange) after heating at 80 °C for 8 h. Bottom: cage **3** (red) and dianiline **A** (green) prior to heating. The reverse experiment (cage **1** ligand displacement by dianiline **C**) showed no change in the spectrum even after heating for 24 h.

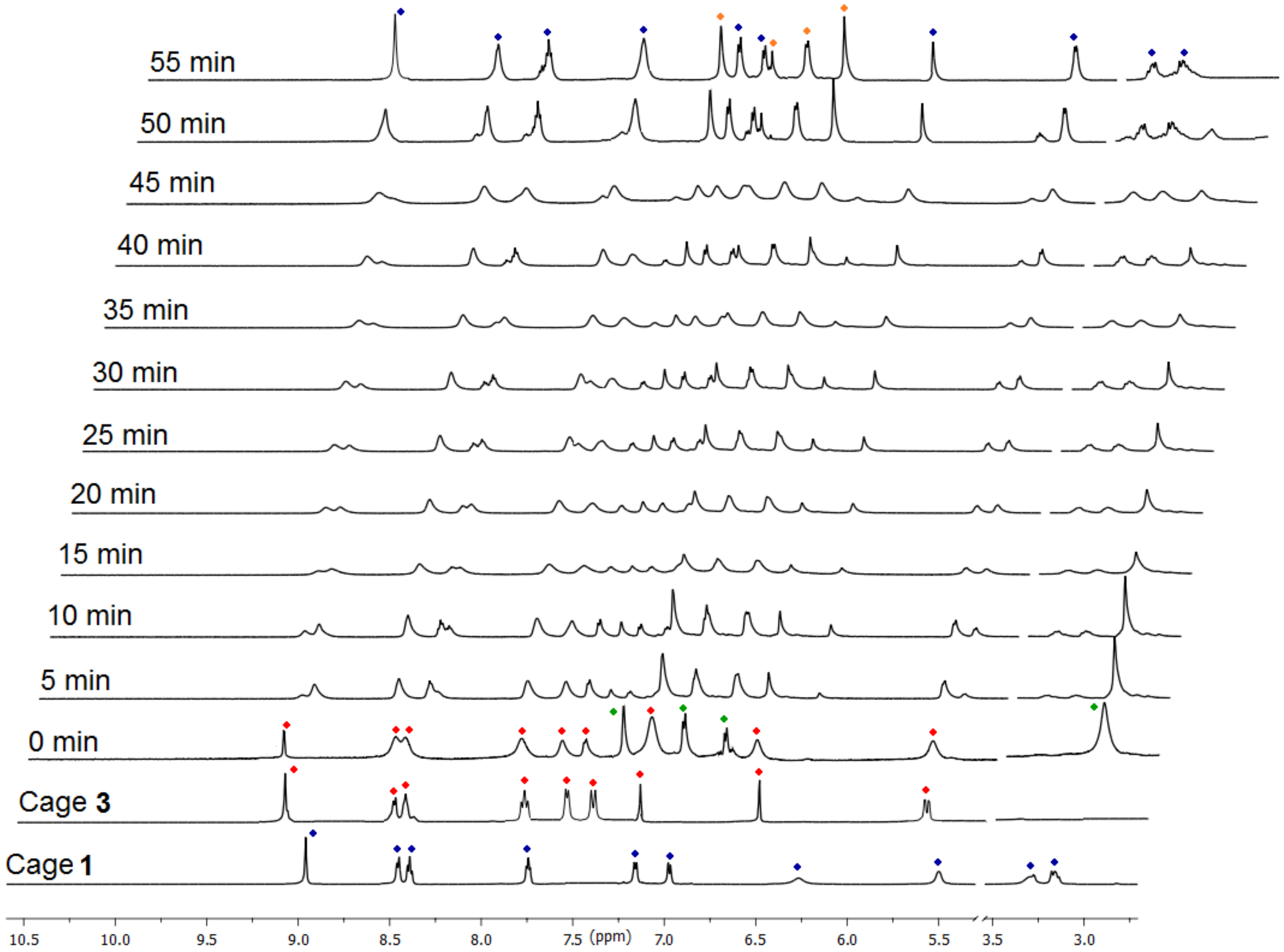

Figure S29: ^{1}H NMR spectra of displacement reaction between cage **3** and dianiline **A** (CD_3CN, 600 MHz, 343 K).

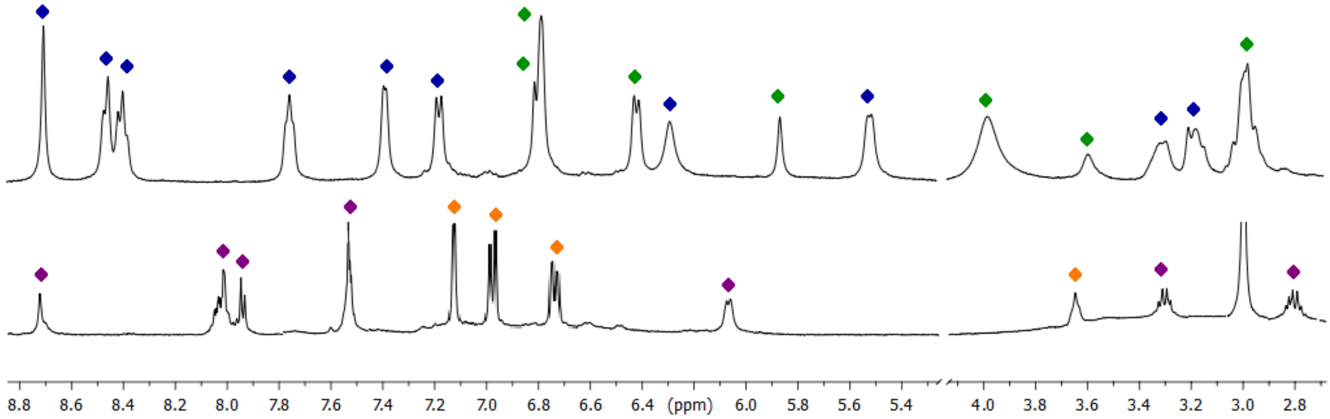

Figure S30: ^{1}H NMR spectra of displacement experiment between cage **2** and dianiline **A** (CD_3CN, 400 MHz, 298K). Top: cage **1** and displaced Dianiline **B** after heating at 80 °C for 8 h. Bottom: cage **2** and dianiline **A** prior to heating. The reverse experiment (cage **1** ligand displacement by dianiline **B**) showed no change in the spectrum even after heating for 24 h.

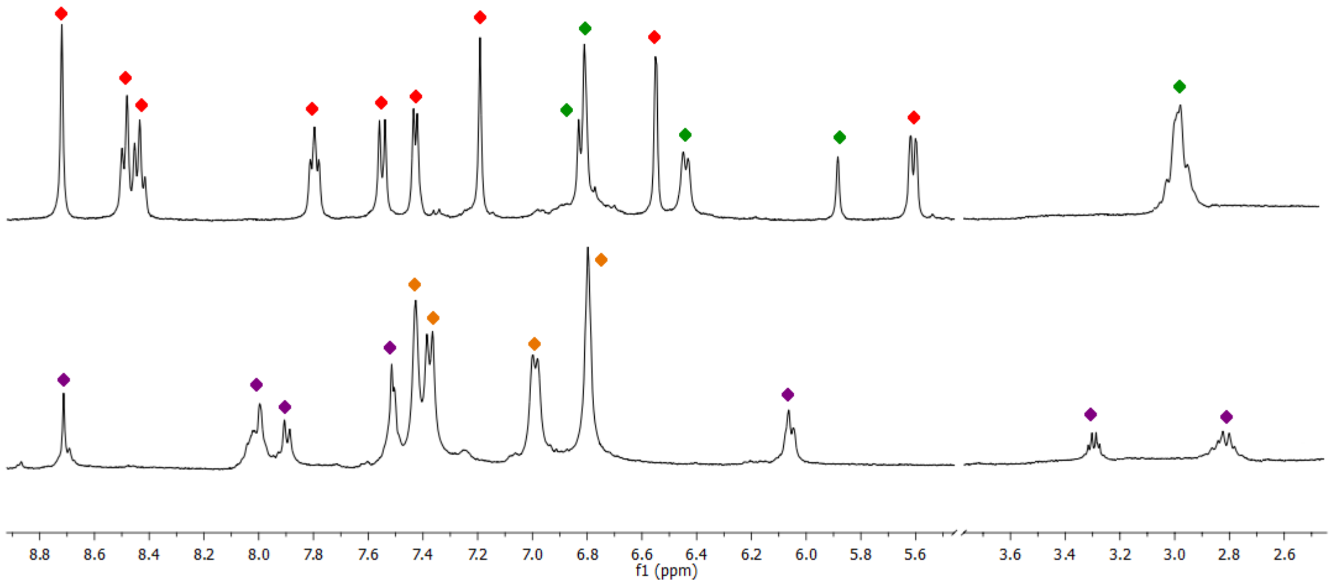

Figure S31: ^{1}H NMR spectra of anhydrous displacement experiment between cage **2** and dianiline **C** (CD_3CN, 400 MHz, 298K). Top: cage **3** and displaced dianiline **B** after heating at 80 °C for 8 h. Bottom: cage **2** and dianiline **C** prior to heating. The reverse experiment (cage **3** displacement by dianiline **B**) showed no change in the spectrum even after heating for 24 h.

References

1) M. C. Young, A. M. Johnson and R. J. Hooley, *Chem. Commun*, 2014, **50**, 1378-1380.
2) A. D. Becke, *J. Chem. Phys.* 1997, **107**, 8554-8560.
3) S. Grimme, *J. Comput. Chem.* 2006, **16**, 1787-1799.
4) W. J. Hehre, R. Ditchfield, and J. A. Pople, *J. Chem. Phys.* 1972, **56**, 2257-2261.
